# Multiomic data analyses unveiled a novel phenol-soluble modulin that induces trapping of extracellular lipases at the surface of *Staphylococcus aureus* cells

**DOI:** 10.1128/mbio.01856-25

**Published:** 2025-09-29

**Authors:** Ane Muruzabal-Galarza, Arancha Catalan-Moreno, Pedro Dorado-Morales, Coral García-Gutiérrez, Miriam Torguet, Isabelle Caldelari, Carlos J. Caballero, Jaione Valle, Alejandro Toledo-Arana

**Affiliations:** 1 Instituto de Agrobiotecnología (IdAB), Consejo Superior de Investigaciones Científicas (CSIC)-Gobierno de Navarra, Mutilva, Navarra, Spain; 2 Université de Strasbourg, CNRS, Architecture et Réactivité de l’ARN, UPR9002 27083, Strasbourg, France; National Institutes of Health, Bethesda, Maryland, USA

**Keywords:** bacterial small proteins, dark proteome, leader peptides, 5’UTRs, PSMs, extracellular lipases, biofilm, protein aggregates, *Staphylococcus aureus*

## Abstract

Small proteins encoded by small open reading frames are often overlooked by annotation algorithms and consequently missing from genome databases. Here, by combining multiomic approaches, we found 27 novel small proteins in *Staphylococcus aureus*, one of the most relevant pathogens worldwide. To better understand the importance of small proteins associated with *S. aureus* virulence factors, we focused on two small proteins encoded in the former 5′- and 3′-untranslated regions of the *sal1* mRNA, respectively. SAL1 is one of the two extracellular lipases involved in *S. aureus* pathogenesis. Both small proteins were predicted as amphipathic helices similar to phenol-soluble modulins (PSMs) and were highly conserved in members of the genus *Staphylococcus*. Our results showed that the *sal1* upstream small protein, LspU, was exported through the same transporters as PSMs, positioning LspU as a putative novel member of the PSM family. Interestingly, constitutive LspU expression induced biofilm structures and produced extracellular amyloidogenic protein aggregates composed of LspU, lipases, and PSMs. Although mutating the PSM-encoding genes reduced said aggregates, it had no impact on lipase trapping at cell surfaces. Altogether, these findings suggest that *S. aureus* may be using LspU to avoid dispersion of lipases during infections and help localize efforts at the infection site.

**IMPORTANCE:**

Bacterial small proteins shorter than 50 amino acids are becoming increasingly recognized as key players for many cellular processes. However, since computational algorithms commonly disregard proteins of this size, most small proteins are not found in genome annotations. In this study, we used genome-scale multiomic analyses to unveil more than 30 novel small proteins, updating the catalog of these proteins in *Staphylococcus aureus*, one of the most relevant bacterial pathogens in hospitals. Among them, we functionally characterized the small protein LspU as a novel member of the phenol-soluble modulin (PSM) family. PSMs are involved in cell toxicity, biofilm structure, and virulence. We showed that LspU induces the formation of extracellular protein aggregates that trap lipases at cell surfaces, which may be a useful strategy to avoid dispersion of extracellular lipases and provide lipase activity at the infection site.

## INTRODUCTION

Bacterial small open reading frames (sORFs) are known for encoding small proteins less than 50–100 amino acids (aa) in length (1–3). The relevance of small proteins for bacterial physiology, environmental adaptation, and pathogenesis is only beginning to emerge. Recently, the combination of optimized bioinformatic workflows and experimental techniques such as genome-wide ribosome profiling (Ribo-seq) and/or shotgun proteomics of several bacterial models has revealed that every bacterium produces numerous small proteins (1, 4–8). Considering that the number of bacterial genomes available in public databases has increased exponentially in the last decade, the expected repertoire of bacterial small proteins is huge. However, automated genome annotations overlook sORFs (9) and, thus, impose a significant bias that prevents generating knowledge on the potential functions of the small proteins. Moreover, several translated sORFs have been identified in formerly denoted intergenic regions and/or non-coding regions, including small regulatory RNAs and untranslated regions (UTRs) of mRNAs (1, 8, 10), leading to a debate on the redefinition of non-coding RNA regions and a rethinking of the understanding of gene organization. sORFs found within former 5′- and 3′-UTRs are known as upstream ORFs (uORFs) and downstream ORFs (dORFs), respectively (5, 11, 12). On the one hand, the translation of uORFs has been associated with leader peptides, which modulate the expression of the main ORF in response to specific metabolites, either by stalling ribosomes and/or modulating the level of their mRNAs (13). On the other hand, although dORFs have also been shown to affect the expression of the main ORF (12), their roles in prokaryotes remain unknown.

Aside from these examples, other roles have been described but only for a reduced number of identified small proteins. For instance, some of them can recruit larger proteins to membranes, stabilize protein complexes, modulate membrane activity, regulate cell division, or induce cell death (3–6, 14). A large percentage of these small proteins tend to be hydrophobic and, as a result, embedded in the bacterial membrane. As a consequence, small proteins have been proposed as promising therapeutic agents to deal with bacterial infections caused by multidrug-resistant (MDR) pathogens (15). Moreover, synthetic peptides derived from natural small proteins with antibacterial or antivirulence properties could be easily manufactured due to their small size and could be used as novel antimicrobial molecules (16).

*Staphylococcus aureus* is one of the most relevant MDR pathogens worldwide, classified in the ESKAPE group that includes a list of bacterial species (*Enterococcus faecium*, *S. aureus*, *Klebsiella pneumoniae*, *Acinetobacter baumannii*, *Pseudomonas aeruginosa*, and *Enterobacter* species) for which new antimicrobial development is urgently needed (17). The characterization of novel small proteins might contribute to such a challenge.

Recently, novel *S. aureus* small proteins (SaSPs) have been identified by applying multiple global approaches, including bioinformatic predictions, optimized mass spectrometry analyses, and Ribo-seq (18–23). Although most of the SaSP functions remain largely unknown, those that have been functionally characterized so far are involved in pathogenesis, stress response, biofilm formation, and toxin-antitoxin (TA) systems.

The paradigm of small proteins in *S. aureus* relies on the phenol-soluble modulins (PSMs), which are a family of amphipathic, α-helical secreted small proteins. Multiple roles have been described for PSMs, including the capacity to produce eukaryotic cell lysis, stimulate inflammatory responses, and contribute to biofilm structure, among others (24). PSMs are classified into two classes: α-type (PSMα1 to PSMα4 and δ-toxin), which are 20–25 aa in length, and β-type (PSMβ1 and PSMβ2), with a size of about 43–45 aa (24). In addition, other SaSPs have been functionally characterized. The cold shock proteins (CSPs) are formed by the 66 aa paralogs CspA, CspB, and CspC. CSPs are involved in staphyloxanthin production, response to cold and oxidative stresses, and biofilm formation (25–28). The *S. aureus* clumping regulator ScrA is an 88 aa membrane protein encoded within the small RNA transcript tsr37. ScrA modulates bacterial aggregation by influencing the activity of the two-component system SaeRS (29, 30). The *ilvD* leader sequence encodes a uORF that produces a 26 aa SaSP rich in branched-chain amino acids (BCAAs; isoleucine, leucine, and valine), which works as cis-acting leucine-responsive attenuator to modulate endogenous BCAA biosynthetic genes in response to exogenous BCAA availability (31). *S. aureus* TA systems, like SprA1/SprA1AS and SprG/SprF (TA type I), and MazEF and YefM-YoeB (TA type II), include stable toxic small proteins that, when triggered, impact various metabolic processes, inducing slow growth and/or cell death (32–35). A TA system activation might contribute to environmental adaptation and microbial defense (36).

In this study, we have updated the catalog of SaSPs by combining RNA-seq, Ribo-seq, and proteomic analyses and identified 90 SaSPs smaller than 50 aa, 27 of which were missing from public databases. To investigate the importance of small proteins that could play a role in *S. aureus* pathogenicity, we focused on SaSPs that were genomically associated with virulence genes. Among them, we identified two conserved 21 aa long SaSPs located within the former 5′- and 3′-UTRs of the *sal1* mRNA. The *sal1* gene encodes the *S. aureus* lipase 1 (SAL1), one of the two extracellular triacylglycerol esterases, which are important virulence factors involved in *S. aureus* pathogenesis (37). Interestingly, both SaSPs associated with the *sal1* gene showed similar features to PSMs, and the constitutive expression of the *sal1* 5’UTR-encoded SaSP induced the trapping of active lipases into biofilm structures.

## RESULTS

### Discovery of novel *S. aureus* small proteins by combining multiomic analyses

To identify novel sORFs and validate the expression of the sORF-encoded small proteins in *S. aureus*, we performed total RNA-seq, Ribo-seq, and shotgun proteomics analyses in the *S. aureus* 15981 strain, a clinical isolate from an otitis infection (38). We obtained total RNA, polysome, and protein extracts from bacterial cultures grown in a rich medium at environmental- and host-related temperatures (22°C and 37°C, respectively) until the early exponential phase (OD_600_ = 0.4). To maximize the protein pool, we also included extracellular and membrane protein extracts. We then performed RNA-seq, Ribo-seq, and mass spectrometry analyses, respectively, as indicated in Materials and Methods. For the identification of small proteins, we correlated the resulting multiomic data with a custom file including all six open reading frames smaller than 50 aa and their corresponding upstream sequence regions. Note that the proteomic approach was not sensitive enough to detect small protein candidates smaller than 50 aa. As a result, the identification of small proteins was mainly based on sORF candidates that presented Ribo-seq signals on the putative ribosome binding sites (RBS) regions (>5 reads in at least one biological replicate). Then, the small protein candidates were manually curated by visualization of the obtained multiomic data track signals in a web server based on JBrowse (39), available at https://rnamaps.unavarra.es/. To facilitate data curation, we loaded the browser with three different genome annotation files including the ones generated by the NCBI (https://www.ncbi.nlm.nih.gov/nuccore/NC_007795.1/), the Bacterial and Viral Bioinformatics Resource Center (BV_BRC; https://www.bv-brc.org/view/Genome/93061.5) (40, 41), and the Bakta web application (https://bakta.computational.bio). The latter detects and annotates small proteins using a custom extraction and filter workflow for sORFs (42). In addition, we also complemented the web browser with additional tracks containing information from previous studies, including the transcriptomic sequencing of *S. aureus* 15981 grown in trypticasein soy broth (TSB) (43), the mapped transcriptional start sites (TSSs) of the *S. aureus* Sc-01 derivative strain (strain O) (44), Ribo-seq data from *S. aureus* JE2 grown in TSB and minimal medium (45), and shotgun proteomic results of extracellular vesicles purified from *S. aureus* CICC 10384 (46). As a result, the workflow allowed us to identify 90 SaSPs smaller than 50 aa, which were further analyzed. The Table S1 shows the coordinate positions of the SaSPs in the reference genome, their protein and RBS region sequences, annotations in public databases, putative function (if known), presence in databases as a positive search by blastP, predicted transmembrane domains and subcellular localization, locus information, Ribo-seq signals, and references (Table S1). In summary, 36 out of the 90 SaSPs were not present in the *S. aureus* NCTC 8325 genome annotations from the NCBI and BV-BRC databases nor predicted by the Bakta web application (Fig. S1). By running a batch blastP analysis using the SaSPs sequence list, we revealed that 57 SaSPs had at least one similar protein annotated in a *S. aureus* genome, reducing to 27 the number of SaSPs with no available information at public databases. In addition, a TMHMM 2.0 analysis helped us predict the presence of a transmembrane domain for 18 SaSPs as well as the localization inside and outside of the cells for 35 and 37 out of 90 SaSPs, respectively (Table S1).

**Fig 1:**
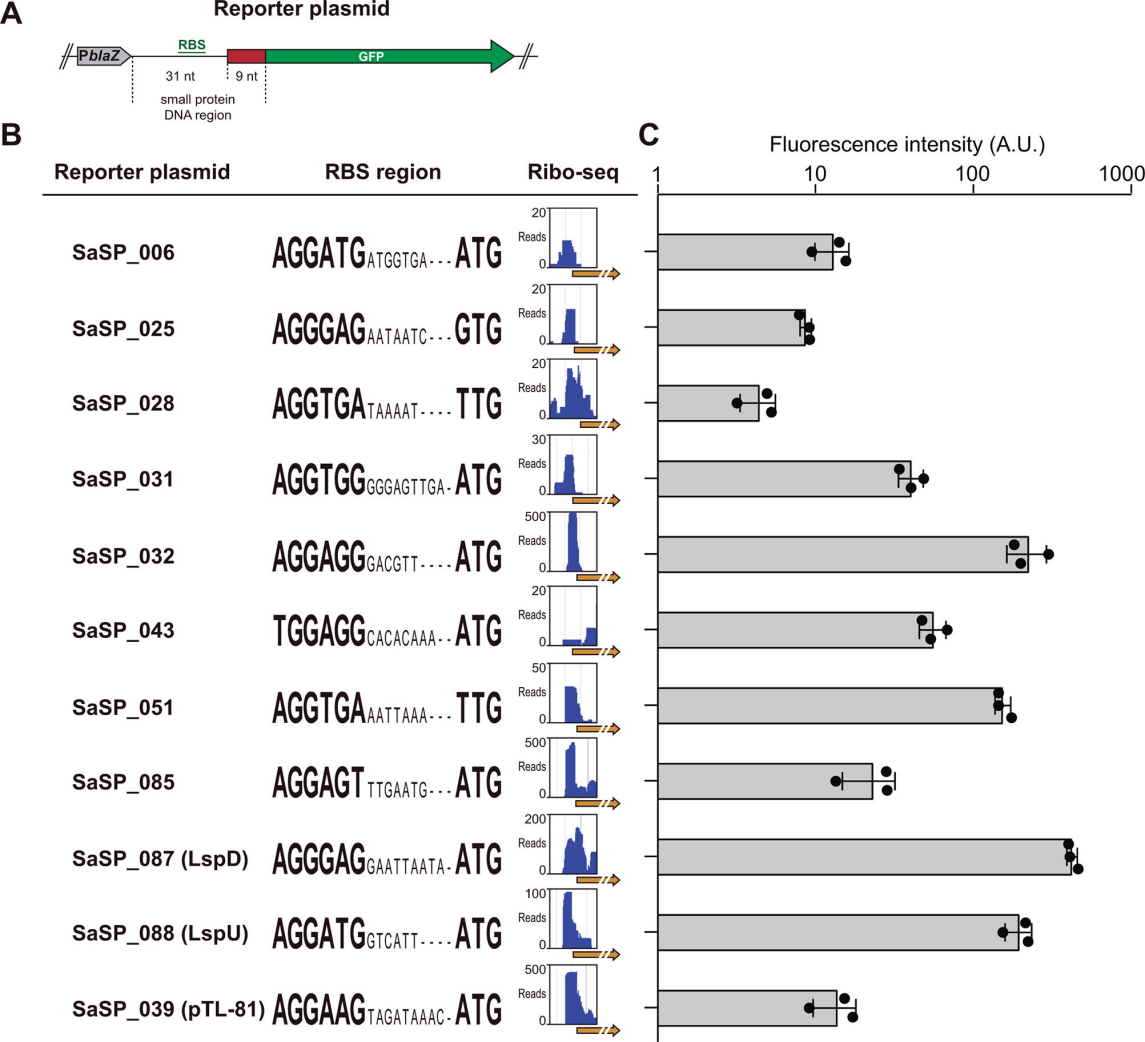
Validation of small protein expression by fluorescent reporter gene fusions. (**A**) Schematic representation of the translational fluorescent reporter plasmids to evaluate small protein expression. Each selected small protein DNA region, including the first three codons, alongside 31 nt upstream of the translation initiation site (including the putative RBS), was fused in-frame with the ATG-less *gfp* gene. (**B**) Plots showing the number of sequencing reads from Ribo-seq analysis mapping to the corresponding RBS region. RBS and start codons are highlighted. (**C**) Bar plots showing fluorescence levels produced from strains carrying the translational fluorescent reporter plasmids. Gray bars represent the mean and SD of GFP fluorescence measured from three independent biological replicates.

Since the proteomic analysis was not sensitive enough to detect the production of SaSPs, we validated the expression of a subset of 10 examples through translational fluorescent reporter gene fusions. For this purpose, we selected candidates encoded in small RNA transcripts (SaSP_006, 032, 043, and 051), 5′-UTRs (SaSP_025 and 088), 3′-UTRs (SaSP_085 and 087), and operons (SaSP_028 and 031; Table S1). Specifically, the first three codons of each sORF alongside 31 nucleotides (nt) upstream of the translation initiation site, which included the putative RBS, were fused in-frame with an ATG-less green fluorescent protein (*gfp*) gene (Fig. 1A). We then electroporated the resulting plasmids into the *S. aureus* 15981 strain and analyzed the GFP expression of the resulting strains by monitoring fluorescence emission in the BioTek Synergy H1 microplate reader. As a positive control, we included a plasmid carrying the *sbrB-gfp* fusion (47). The SbrB small protein (SaSP_039; Table S1) has been validated in previous studies (47, 48), and as well as the selected small proteins, it showed Ribo-seq signals at the RBS regions (Fig. 1B). As a background control, an *sbrB* mutant carrying a premature stop codon was included. Figure 1B shows that all plasmids carrying the small protein reporter fusions produced GFP, indicating that the novel sORFs were actively translated (Fig. 1). Altogether, our multiomic analyses using in-house and publicly available data unveiled the expression of novel small proteins from the *S. aureus* dark proteome.

### The *sal1* mRNA produces two highly conserved amphipathic small proteins similar to PSMs

**Fig 2:**
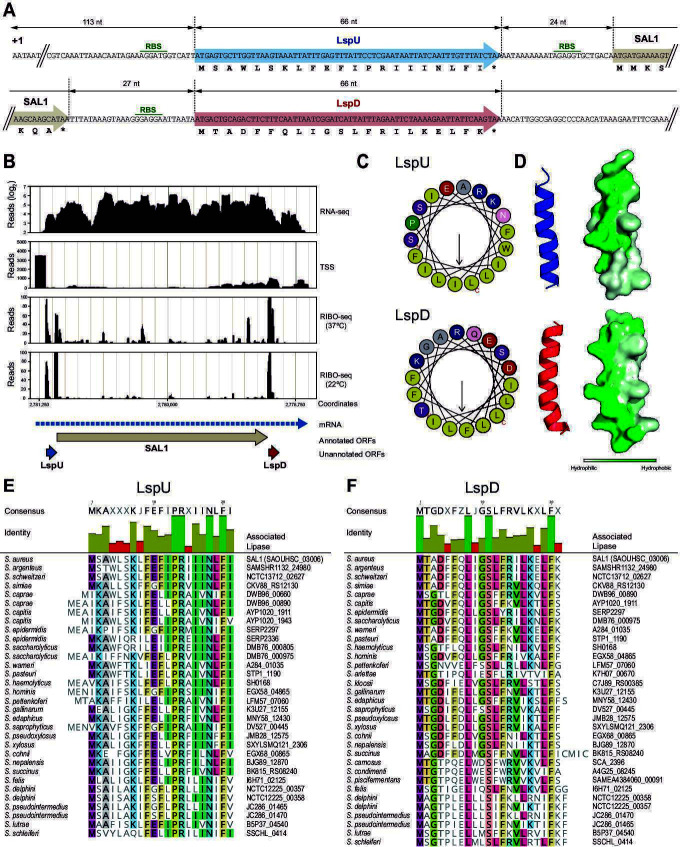
The *sal1* mRNA encodes two novel amphipathic small proteins. (**A**) DNA sequences of the 5′ and 3′ regions of the *sal1* mRNA, including the LspU and LspD sORFs. LspU and LspD aa sequences are represented below the corresponding codons. The distances between the different features are indicated in nt. The RBSs are marked. (**B**) JBrowse images showing data tracks representing the RNA sequencing reads from total RNA-seq, TSS, and Ribo-seq analyses that mapped to the *sal1* gene region. Genomic coordinates corresponding to the *S. aureus* NCTC 8325 reference genome are indicated. The complete transcriptomic maps are available at http://rnamaps.unavarra.es/. Dashed and colored arrows represent the *sal1* mRNA and the LspU, SAL1, and LspD ORFs, respectively. (**C**) Computation of α-helical wheels in LspU and LspD using the program available at http://heliquest.ipmc.cnrs.fr/. LspU and LspD form amphipathic α-helices according to the opposite arrangement of hydrophobic (yellow) vs charged (red and blue) and hydroxyl (purple) aa. (**D**) Modeling of LspU and LspD structures using AlphaFold2 (https://alphafoldserver.com) (51). Prediction of the hydrophobicity profile of the protein surface, where green indicates most hydrophobic regions, and white indicates most hydrophilic regions, generated with UCSF chimeraX (52). (**E**) LspU and (**F**) LspD multiple sequence alignments of small protein orthologs from members of the genus *Staphylococcus*. The identity of each aa is represented by bars. Consensus sequences are represented at the top. The gene name for the lipase gene associated with the corresponding small protein is indicated on the right of each sequence.

The success of *S. aureus* infections relies on the production of a broad array of virulence factors (49). Therefore, we focused on SaSPs in the vicinity of virulence genes, expecting a functional relationship and, thus, SaSPs’ implication on pathogenesis. We found two novel sORFs (SaSP_087 and SaSP_088; Fig. 1B; Table S1) that were located near the *sal1* gene (SAOUSC_03006), which encodes SAL1 (Fig. 2A). SAL1 is one of the two extracellular triacylglycerol esterases involved in *S. aureus* virulence (37). In addition to the expected Ribo-seq signal peaks that indicated the translation of SAL1, additional peaks were found at both the 5′ and 3′ regions of the *sal1* gene, where no other ORFs were previously annotated (Fig. 2B). Such Ribo-seq translation peaks were located upstream of two sORFs, at regions rich in G bases, indicating the presence of potential RBSs (Fig. 1B and 2A), which were further validated by GFP reporter fusions (Fig. 1B). According to the total RNA-seq data, the boundaries of both sORFs were mapped inside the *sal1* mRNA, indicating that it was a polycistronic operon that encoded three proteins under the control of the *sal1* promoter. Both small proteins were 21 aa long, and we named them LspU and LspD for lipase-associated small protein, upstream and downstream of SAL1, respectively. On the one hand, the upstream small ORF (*sal1* uORF, *lspU*) had its start codon located 113 nt downstream from the TSS of the *sal1* mRNA and its stop codon 24 nt upstream from the SAL1 start codon. On the other hand, the downstream small ORF (*sal1* dORF, *lspD*) had its start codon located 27 nt away from the SAL1 stop codon (Fig. 2A). Interestingly, the Bakta application annotated LspD as a putative phenol-soluble modulin PSMα3. Note that LspD was previously identified by Nikoleit et al. when characterizing the *S. aureus sal1* gene, formerly known as *geh* (50). However, it was never annotated in the reference *S. aureus* genomes from the public NCBI and BV_BRC genome databases.

SAL1 shares 43.6% of protein sequence identity with SAL2 (SAOUHSC_00300), the other paralogous extracellular lipase of *S. aureus*. Although the *sal2* mRNA also showed strong Ribo-seq signals corresponding to SAL2 translation, no signals that indicated sORFs in the *sal2* 5′-UTR or 3′-UTR were found (http://rnamaps.unavarra.es).

To predict the secondary structure of LspU and LspD, we used the Jpred 4, HeliQuest, and C-QUARK servers (53–55). According to the outputs from these servers, both LspU and LspD were predicted as amphipathic helices, which have hydrophobic and hydrophilic/charged residues situated on opposite faces of the predicted helix (Fig. 2C). We further confirmed this structural organization by running predictions on AlphaFold server (https://alphafoldserver.com) (51) (Fig. 2D), which provided structures resembling those of the α-class PSMs (56, 57).

Lipases are widely distributed among species of the genus *Staphylococcus* and other bacterial pathogens, suggesting that the *sal1*, *lspU*, and *lspD* association may be extended beyond *S. aureus*. To verify this hypothesis, we performed comparative sequence analyses. Since *lspU* and *lspD* genes were not annotated in public protein databases, we carried out sequence comparisons using tblastn from the BLAST application of NCBI (https://blast.ncbi.nlm.nih.gov/Blast.cgi). The tblastn output showed that the LspU and LspD small protein sequences were present in 26 and 31 different *Staphylococcus* species, respectively (Fig. 2E and F; Fig. S2), indicating that they were both highly conserved among members of the genus *Staphylococcus*. Interestingly, the genomes of *Staphylococcus delphini* and *Staphylococcus pseudointermedius* encoded two copies of *lspU* and *lspD. Staphylococcus caprae*, *Staphylococcus capitis*, *Staphylococcus epidermidis,* and *Staphylococcus saccharolyticus* carried two copies of *lspU* but only one of *lspD,* and *Staphylococcus arlettae*, *Staphylococcus kloosii*, *Staphylococcus carnosus*, *Staphylococcus condimenti,* and *Staphylococcus piscifermentans* a single copy of *lspD* (Fig. 2E and F; Fig. S2). Next, considering that the number of genes encoding extracellular lipases was variable among *Staphylococcus* species, we investigated whether the *lsp* sORFs were co-transcribed with a specific lipase. Thus, extracellular lipase protein sequences from the species having orthologous LspU/D were clustered with Geneious Prime application using the SAL1 and SAL2 protein sequences as references. Fig. S2 shows that *lspU/D* were associated with genes encoding SAL1 orthologs in most of the *Staphylococcus* spp. (Fig. S2).

Altogether, these results confirmed that the *sal1* mRNA produced, in addition to SAL1, two highly conserved small proteins, LspU and LspD, with similar features to PSMs.

### Mutation of *lspU* did not affect SAL1 translation levels

As aforementioned, uORFs often modulate the expression of main ORFs through different mechanisms (13). Following this idea, we focused on further analyzing the biological function of the *sal1* uORF *lspU*. Therefore, we first investigated whether the translation of the *lspU* uORF could modulate the expression of the main gene *sal1*. To do that, we generated translational *sal1-gfp* fusion reporters that carried either a wild type (WT) or a mutated version of the *lspU* gene. In the latter, this consisted of a single base substitution that produced a premature stop codon for *lspU* (Fig. S3A). To ensure that the base substitution effectively inhibited the LspU production, we constructed translational *lspU-gfp* fusion reporters that carried the wild type and the *lspU* mutated version, respectively. The expression of each plasmid was controlled by the native *sal1* promoter (Fig. S3A). We then used the constructed plasmids to electroporate *S. aureus* 15981 strain and grew the generated strains until the exponential growth phase. To address the protein expression levels, we ran western blot analyses from the protein extracts and found that, although the stop codon substitution impaired the production of LspU-GFP, it had no effect on the SAL1-GFP protein levels, suggesting non-regulatory roles for LspU in the tested conditions (Fig. S3B).

### The constitutive expression of LspU reduced lipase activity in supernatants and promoted cell aggregation

**Fig 3:**
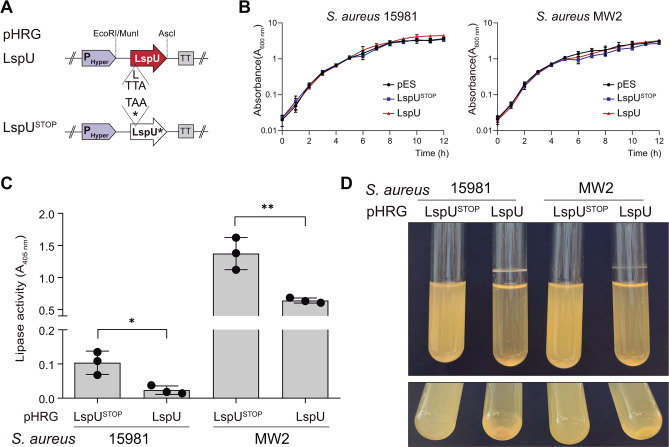
Constitutive expression of LspU reduces lipase activity in supernatants and increases biofilm formation and bacterial aggregation. (**A**) Schematic representation of the constructed pHRG-LspU and pHRG-LspU^STOP^ plasmids carrying the native and mutated versions of *lspU* uORF, respectively. The base substitution (TTA for TAA) made in the fifth codon to generate a stop codon is indicated (for strains and plasmids details see the supplemental material). (**B**) Growth curves of the *S. aureus* 15981 and MW2 strains carrying the pHRG-LspU and pHRG-LspU^STOP^ plasmids in MH broth. (**C**) Lipase activity in culture supernatants from the *S. aureus* 15981 and MW2 strains carrying the pHRG-LspU and pHRG-LspU^STOP^ plasmids. The lipase activity was assayed on spent culture supernatants from cells grown in MH for 16 h. The release of p-nitrophenol from p-nitrophenyl butyrate was measured by spectrophotometry at 405 nm. Bar plots represent the mean and SD of *A*_405_ measured from three independent biological replicates. Asterisks represent statistical significance (*, *P* < 0.05; **, *P* < 0.005; *t*-test); ns, not significant. (**D**) Phenotypic comparison of the *S. aureus* 15981 and MW2 strains carrying the pHRG-LspU and pHRG-LspU^STOP^ plasmids. Biofilm formation and bacterial aggregation phenotypes were observed after incubation for 16 h at 200 rpm and 37°C in glass tubes containing 5 mL of MH broth. Representative images from the triplicates are shown.

Since LspU translation had no apparent relationship with SAL1 production in the tested conditions, we then analyzed the effect of a constitutive expression of LspU on *S. aureus* phenotypes. To this end, we cloned the 5′ region of the *sal1* mRNA, including the *lspU* ORF, under the control of the constitutive P*hyper* SPO1 promoter (58), generating the pHRG-LspU plasmid, which we transformed into the *S. aureus* 15981 strain. As negative controls, we included the pES and pHRG-LspU^STOP^ plasmids, which carried the mutated version of *lspU*, as previously shown (Fig. 3A; Fig. S3A). Note that the clinical *S. aureus* 15981 isolate used to identify the small proteins is defective in the Agr quorum system (59), which modulates the expression of secreted proteins, including SAL1 and SAL2. Consequently, in addition to strain 15981, we also transformed the reference *agr*-positive MW2 strain with the same plasmids. We then grew the resulting strains in Mueller-Hinton (MH) medium to evaluate the growth rate and lipase activity of the supernatants. Figure 3B shows that all strains presented similar growth rates, indicating that the constitutive expression of LspU did not affect cell viability. We also determined the extracellular lipase activity from supernatants of cultures grown in MH broth for 16 h. We used a colorimetric coupled enzyme reaction, which releases p-nitrophenol after lipase hydrolysis of p-nitrophenyl butyrate (60). After measuring the levels of p-nitrophenol at 405 nm, we found the lipase activity to be higher in MW2 than in 15981, indicating that the Agr system is required for full expression of extracellular lipases in the former strain. Nevertheless, the LspU expression significantly reduced the lipase activity in the supernatants of both the 15981 and MW2 pHRG-LspU strains in comparison to controls, 15981 and MW2 pHRG-LspU^STOP^, respectively (Fig. 3C). In addition, when growing said strains in glass tubes, we found that the constitutive expression of LspU induced the formation of a ring over the liquid-air interface, combined with bacterial aggregation at the bottom of the tube, all of which indicated the activation of biofilm formation (Fig. 3D). To characterize the biofilm composition, we treated mature biofilms with either Proteinase K, DNase I, or sodium metaperiodate, which digest biofilms mainly structured by proteins, DNA, or polysaccharides, respectively. Fig. S4 shows that the biofilm ring over the liquid-air interface was eliminated by Proteinase K and unaltered by DNase I and metaperiodate, suggesting that LspU-mediated biofilm structures are mainly protein based (Fig. S4). Altogether, these results suggest that LspU might have a role in modulating lipase activity and promoting cell aggregation and/or proteinaceous biofilm formation, phenotypes that seemed to be strain independent. From this point on, we conducted the remaining experiments using the agr-positive MW2 strain for the sake of simplicity.

### The expression of LspU reduced the levels of SAL1 and SAL2 in culture supernatants

**Fig 4:**
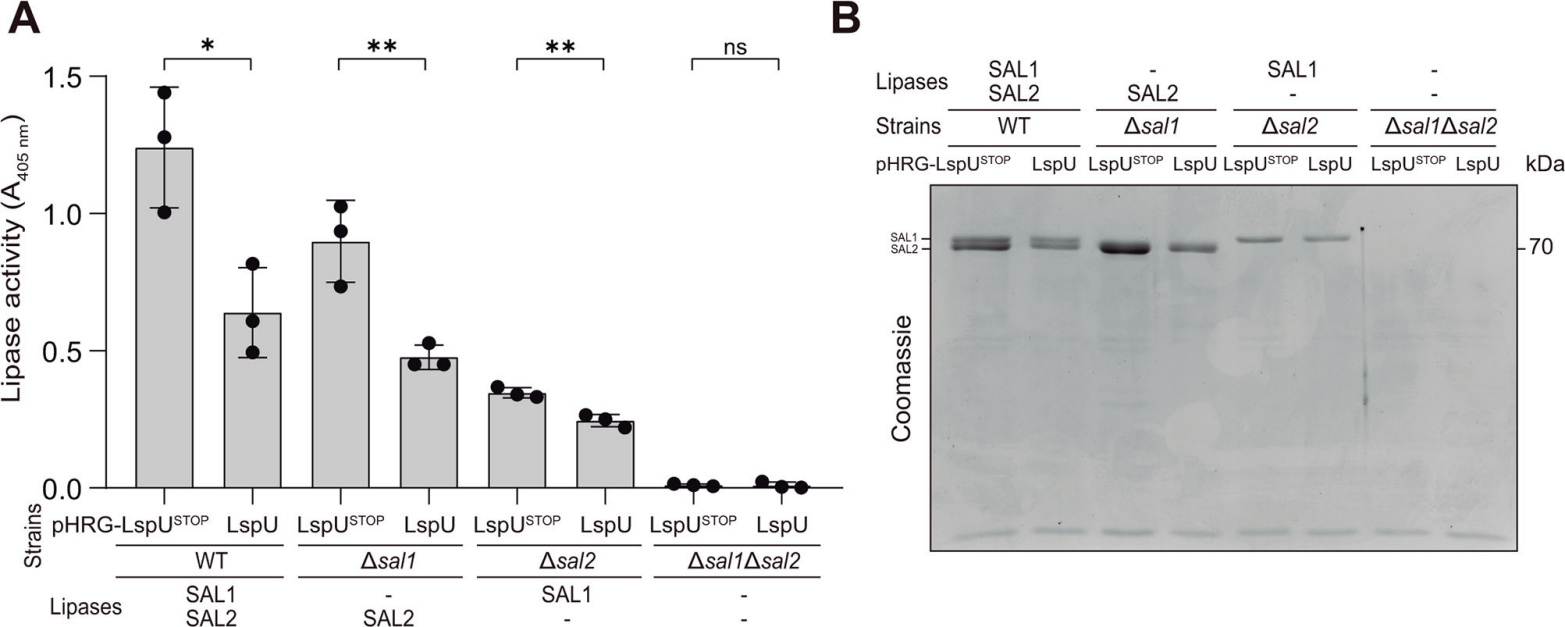
The expression of LspU reduced the SAL1 and SAL2 protein levels and lipase activity in culture supernatants. (**A**) Lipase activity in culture supernatants from *S. aureus* MW2 WT and single and double *sal1* and *sal2* mutant strains carrying the pHRG-LspU and pHRG-LspU^STOP^ plasmids. Lipase activity was assayed on OD_600_ normalized spent culture supernatants from cells grown in MH broth for 16 h at 37°C and 200 rpm. Release of p-nitrophenol from p-nitrophenyl butyrate was measured by spectrophotometry at 405 nm. Bar plots represent the mean and SD of *A*_405_ measured from three independent biological replicates. Asterisks represent statistical significance (*, *P* < 0.05; **, *P* < 0.005; *t*-test); ns, not significant. (**B**) Stained SDS-PAGE separation of protein extract of OD_600_ normalized spent culture supernatants from the same strains grown in MH broth for 16 h at 37°C and 200 rpm. A representative image from biological triplicates is shown.

To investigate whether the inhibition of lipase activity in the supernatants exerted by LspU was specific to SAL1 or if it could also affect SAL2, we constructed single and double *sal1* and *sal2* mutants in the *S. aureus* MW2 strain through allelic replacement, using the pMAD plasmid (61). Next, we transformed the mutants with the pHRG-LspU and pHRG-LspU^STOP^ plasmids. The *sal1* and *sal2* single mutants showed a 25% and 75% reduction in the total culture lipase activity when compared with their wild-type counterpart, respectively (Fig. 4A). This indicated that SAL2 contributed more than SAL1 to the total lipase activity of the supernatant in the tested conditions. The double mutant displayed no lipase activity, indicating that both proteins contributed to the total lipase activity in the supernatants (Fig. 4A). Moreover, a constitutive expression of LspU inhibited lipase activity in the culture supernatants of both SAL1 (Δ*sal2* pHRG-LspU) and SAL2 (Δ*sal1* pHRG-LspU). However, it seemed that the differences in lipase activity reduction were smaller in SAL1 than in SAL2 (Fig. 4A).

The reduction of lipase activity in culture supernatants due to LspU constitutive expression could be explained by LspU directly blocking the lipase enzyme activity and, consequently, diminishing its hydrolysis capacity. Alternatively, LspU might affect the SAL1 and SAL2 export, reducing their protein levels in the supernatants. To gain further insight into the mechanism, we evaluated the SAL1 and SAL2 protein levels in the supernatant by running fractions in 10% polyacrylamide gels, as previously described (60). Two protein bands around 70 kDa, compatible with the sizes of pro-lipases SAL1 and SAL2, were observed in the culture supernatants from the wild-type MW2 strain. No bands at the size of mature lipases were detected, indicating that the total lipase activity was produced by the pro-lipases (Fig. 4). One of the ~70 kDa upper bands disappeared in the single Δ*sal1* mutant, while the lower band could not be detected in the single Δ*sal2* mutant. In the double Δ*sal1* Δ*sal2* mutant, both bands disappeared (Fig. 4B). This confirmed that the ~70 kDa upper and lower bands corresponded to SAL1 and SAL2, respectively. Constitutive expression of LspU in these mutants reduced the levels of both lipase bands in the wild-type and single Δ*sal* mutants (Fig. 4B). This result indicated that the lower lipase activity exerted by LspU expression could be a consequence of a reduction of pro-lipase levels in the culture supernatants (Fig. 4B).

### The constitutive expression of LspU retained active lipases in cell pellet fractions

**Fig 5:**
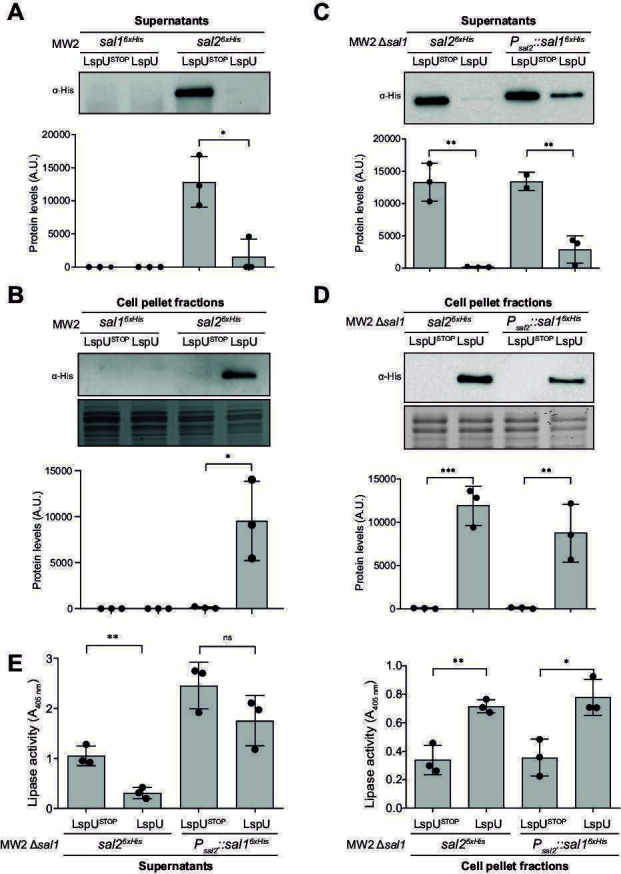
LspU expression led to active lipases retention in cell pellet fractions. Western blots showing the 6×His-tagged SAL1 and SAL2 protein levels in protein extracts of OD_600_ normalized supernatants (**A and C**) and protein concentration normalized cell pellet (**B and D**) fractions from the indicated strains carrying the pHRG-LspU and pHRG-LspU^STOP^ plasmids grown in MH broth for 16 h at 37°C and 200 rpm. Membranes were developed using anti-His antibodies and a bioluminescent kit. Stained gel portions are included as loading controls. Bar plots represent the mean and SD of SAL1^6×His^ and SAL2^6×His^ levels from three independent biological replicates, which were determined by densitometry of western blot bands using ImageJ (https://imagej.nih.gov/ij/). Representative images from biological triplicates are shown. (**E**) Bar plots showing the lipase activity in supernatants and cell pellet fractions from strains carrying the pHRG-LspU and pHRG-LspU^STOP^ plasmids. The lipase activity was assayed on spent culture supernatants or cell pellets harvested by centrifugation after growth in MH broth for 16 h at 37°C and 200 rpm. The release of p-nitrophenol from p-nitrophenyl butyrate was measured by spectrophotometry at 405 nm. Asterisks represent statistical significance (*, *P* < 0.05; **, *P* < 0.005; ***, *P* < 0.0005; *t*-test); ns, not significant.

To further understand how LspU reduced the SAL levels when constitutively expressed, we chromosomally labeled both *sal1* and *sal2* with the 6×His tag at the C-terminus of each ORF by allelic replacement, generating the MW2 *sal1^6×His^* and MW2 *sal2^6×His^* strains. We then evaluated the levels of each of the lipases (SAL1^6×His^ and SAL2^6×His^) in the supernatants by western blot. In agreement with our previous results, we confirmed that SAL2^6×His^ had its levels reduced when LspU was constitutively expressed, confirming the observed results with the untagged strains (Fig. 5A). Unfortunately, SAL1^6×His^ could not be detected in this condition unless much higher concentrations of total protein were loaded (data not shown). When analyzing the protein extract derived from cellular pellets of the aforementioned strains, we revealed higher levels of SAL2^6×His^ in the LspU-expressing strain in comparison with the negative control (Fig. 5B). This suggested that LspU promoted the accumulation of SAL2 lipase in the cell pellet fraction. No visible bands could be detected in cell pellet fractions from the MW2 *sal1^6×His^* strain, even when we loaded with higher protein concentrations. This might be explained by the expression of SAL1, which was produced at much lower levels than SAL2, in the evaluated conditions. As a result, this experiment could not rule out whether LspU retained both lipases to cell fractions or SAL2 only. To gain further resolution, we constructed a strain in which the *sal2* ORF was replaced by that of *sal1* and included a poly-histidine tag at the C-terminal so that SAL1^6×His^ was expressed under the control of the *sal2* promoter (P*_sal2_*). This strategy would allow us to produce similar levels to those of SAL2 and reassess the effect of LspU under the new conditions. The MW2 P*_sal2_::sal1^6×His^* Δ*sal1* strain was generated by allelic replacement. As a control, the MW2 *sal2^6×His^* Δ*sal1* strain was also constructed, which only expressed SAL2^6×His^. When performing western blots with the generated strains, we found that SAL1^6×His^ was produced in comparable amounts to those of SAL2^6×His^ when expressed under the influence of the P*_sal2_* promoter. In this scenario, a constitutive expression of LspU reduced the SAL1 lipase activity by reducing its levels in supernatants, but this effect was lower than the one observed for SAL2^6×His^ (Fig. 5C). In agreement with this, the accumulation of SAL1^6×His^ in the cell-pellet fractions was lower than that of SAL2^6×His^ (Fig. 5D).

Then, we wondered whether the lipase activity was preserved in the cell-pellet fractions. For evaluating this, we measured the lipase activity of the supernatant and cell-pellet fractions. We showed that, in all cases, the lipase activity correlated to the observed levels of lipases present either in the supernatants or the cell fractions, indicating that SAL1 and SAL2 were active in both fractions (Fig. 5E). Altogether, these results indicated that the constitutive expression of LspU accumulated active lipases in cell-pellet fractions, reducing SAL2- and, to a lesser extent, SAL1-mediated lipase activity in *S. aureus* supernatants.

### LspU localizes in cell pellet fractions with lipases

**Fig 6:**
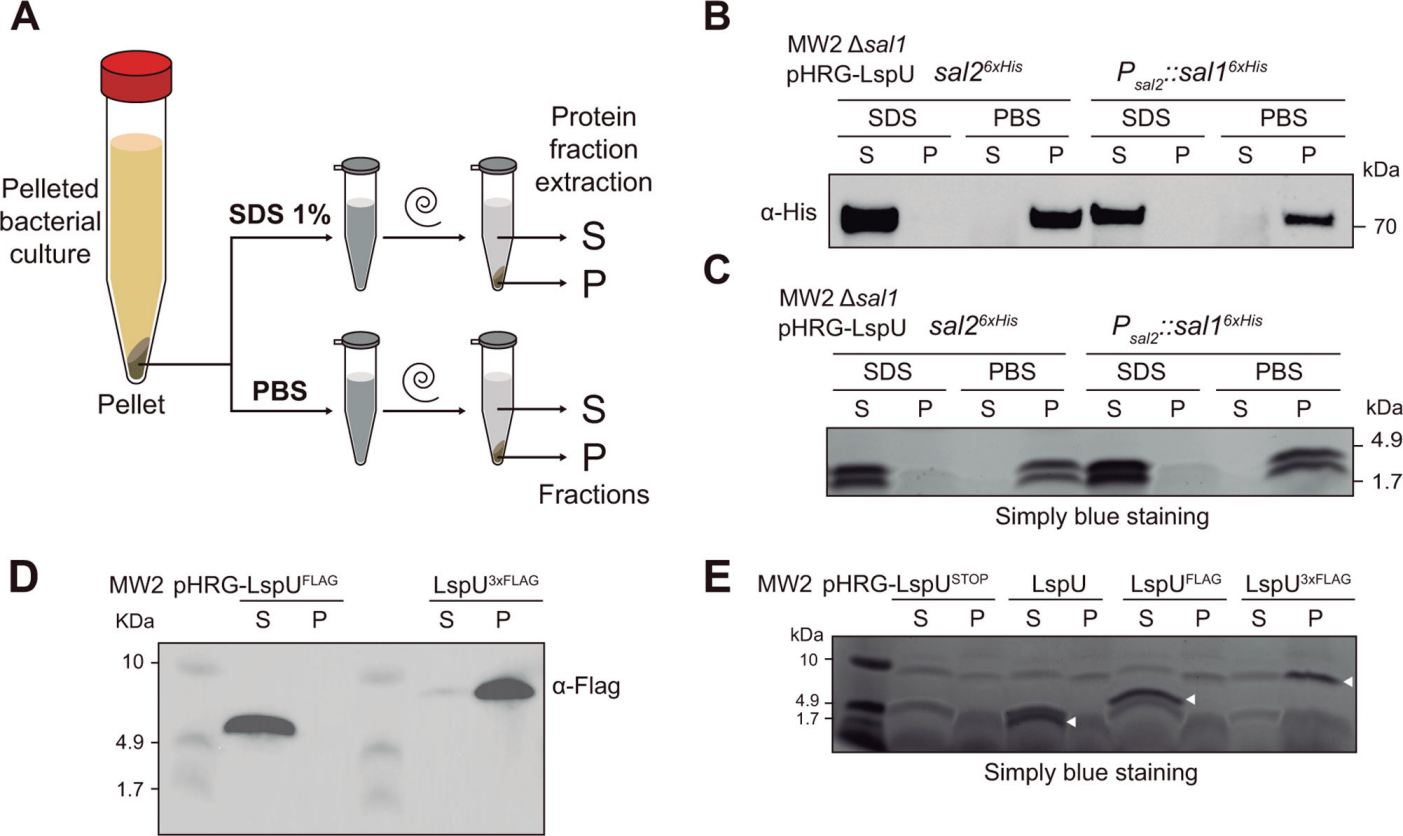
LspU is extracted from cell pellet fractions by 1% SDS. (**A**) Schematic representation of the procedure followed to obtain SDS-soluble protein fractions from cell pellets normalized by OD_600_ of strains grown in MH broth for 16 h at 37°C and 200 rpm. PBS was used as a control. S, soluble and P, pellet fractions after 1% SDS or PBS treatment. (**B**) Western blots showing the 6×His-tagged SAL1 and SAL2 protein levels in protein extracts from the soluble (S) and pellet (P) fractions after treatment of cell pellets with 1% SDS or PBS. Membranes were developed using anti-His and secondary antibodies conjugated with peroxidase and a bioluminescent kit. (**C and E**) Simply blue staining of SDS-PAGE separation of small proteins from the same extracts used in panels B and D, respectively. (**D**) Western blots showing the FLAG- and 3×FLAG-tagged LspU protein levels in protein extracts from the soluble (S) and pellet (P) fractions after treatment of cell pellets with 1% SDS. Membranes were developed using peroxidase-conjugated anti-FLAG antibodies and a bioluminescent kit.

It has been shown that the *S. aureus* cell envelope selectively controls the sorting of several virulence factors, including the pro-lipase and mature lipase SAL2. Specifically, it was described that SAL2 was associated with surface-exposed compartments in the bacterial cell envelope (62). In agreement with these observations, we hypothesized that SAL1 and SAL2, present in the cell-pellet fractions, were being retained in such compartments by the action of LspU. To address this idea, we grew the MW2 *sal2^6×His^* Δ*sal1* and MW2 *P_sal2_::sal1^6×His^* Δ*sal1*strains expressing LspU until the stationary phase. We then obtained cellular pellets by centrifugation that were subjected to SDS treatment, as described by Zheng and colleagues to release lipases (62), or washed with phosphate-buffered saline (PBS) as a control. Total proteins extracted from SDS-soluble fractions (S) and cell-pellet fractions (P) after SDS or PBS treatments were run in an SDS-PAGE, and western blots were performed (Fig. 6A). The results revealed that both SAL1 and SAL2 could be extracted with SDS from the cell pellets (Fig. 6B). This suggested that LspU could promote the trapping of lipases in the cell-pellet fractions.

We hypothesized that LspU could be mediating lipase anchorage to the cell envelope, since amphipathic helices like the one presented by LspU are a common structural motif that often serves as a means for anchoring proteins to cell envelopes (63). This would imply LspU being exported to the same compartment as lipases, and therefore, it would be possible to extract LspU using 1% SDS. Since LspU has an expected size of ~2.5 kDa, we run the soluble and cell-pellet fractions from the previous SDS and PBS treatments in 10–20% Tris-tricine gradient gels to reveal small proteins by SimplyBlue staining. As observed in Fig. 6C, two small protein bands that migrated between the 1.7 and 4.7 kDa markers were visible in the SDS soluble fraction, while retained in cell fractions when treated with PBS. To confirm whether any of those bands corresponded to LspU, we tagged LspU with different epitopes (6×His, 3×FLAG, and FLAG) at the N- and C-terminus. However, we found that neither the FLAG-tagged nor 6×His-tagged LspU small proteins could reproduce the LspU-mediated phenotypes, indicating that tagged versions affected LspU functionality. Despite this, we performed western blots, using the LspU^3×FLAG^ and LspU^FLAG^ versions to corroborate if the tagged LspUs were localized in the same fractions as SAL1 and SAL2 lipases. Interestingly, we found that both tagged LspU versions were retained in the cell pellets; however, only the LspU^FLAG^ could be extracted using 1% SDS, suggesting that native LspU and tagged LspU^FLAG^ were equally localized (Fig. 6D). Further analysis showed that LspU^3×FLAG^ was associated with membrane and cytosolic protein fractions (Fig. S5), indicating that the longer 3×FLAG tag might compromise LspU’s extracellular export. Moreover, since the insertion of tags resulted in LspU versions with higher molecular weight bands, which migrated differently, staining of protein gels indicated that the lower small protein band seen in the non-tagged version of LspU indeed corresponded to LspU (Fig. 6E). Based on these results, we concluded that LspU was retained in the same fractions as lipases, suggesting a possible interaction between both proteins.

### LspU induced the formation of extracellular aggregates that trap lipases

**Fig 7:**
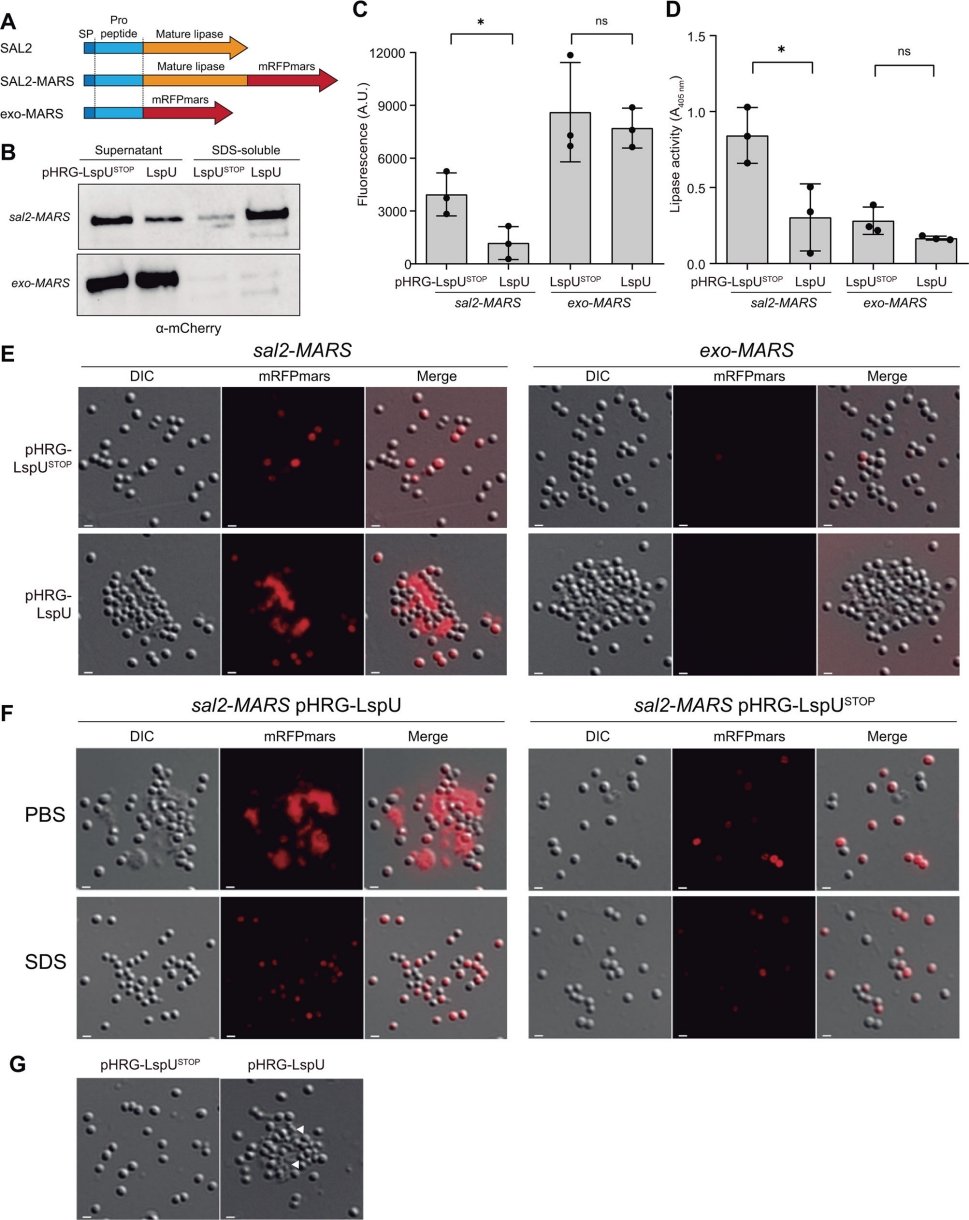
The expression of LspU traps SAL-MARS in protein aggregates. (**A**) Schematic representation of different genetic constructs to generate SAL2-MARS and exo-MARS strains. SAL2-MARS expresses the SAL2 extracellular lipase fused to the codon-optimized RFP variant *mRFPmars* (64), which was inserted in frame with the full length of *sal2* ORF in *S. aureus* MW2. The exo-MARS strain carried the *mRFPmars* gene, which was fused in frame and downstream of the *sal2* pre-pro region to export the red fluorescent protein to supernatants. (**B**) Western blots showing the chimeric red protein levels in the protein extracts from the supernatant and SDS-soluble fractions of the strains SAL2-MARS and exo-MARS carrying the pHRG-LspU and pHRG-LspU^STOP^ plasmids grown in MH broth for 16 h at 37°C and 200 rpm. Membranes were developed using anti-mCherry and secondary antibodies conjugated with peroxidase and a bioluminescent kit. (**C and D**) Bar plots showing red fluorescence (A.U.) and lipase activity (*A*_405_) levels, respectively, in the supernatants of the indicated strains grown in the same conditions as explained before. Asterisks represent statistical significance (*, *P* < 0.05; *t*-test); ns, not significant. (**E**) Representative images of microscopic acquisitions in the differential interference contrast (DIC) and red (mRFPmars) channels of the SAL2-MARS and exo-MARS strains carrying the pHRG-LspU and pHRG-LspU^STOP^ plasmids grown in MH broth for 16 h. Merged DIC and mRFPmars images (Merge) are shown. Scale bars, 1 µm. (**F**) Representative images of microscopic acquisitions in the DIC and mRFPmars channels of the cell pellet fractions after 1% SDS or PBS treatment. (**G**) Representative images of microscopic acquisitions in the DIC channel of MW2 strain carrying the pHRG-LspU and pHRG-LspU^STOP^ plasmids and grown in MH broth for 16 h. Protein aggregates are indicated with white triangles.

To determine if lipases and LspU co-localized to the cell envelope, we aimed at investigating the subcellular localization of both proteins by constructing compatible fluorescent chimeric proteins. On the one hand, we generated a plasmid expressing LspU fused to GFP (pHRG-LspU-GFP). On the other hand, we constructed by allelic replacement an MW2 strain expressing SAL2 fused to the codon-optimized red fluorescent protein (RFP) variant *mRFPmars* (SAL2-MARS) (64), which was inserted in frame before the stop codon of the *sal2* gene in *S. aureus* MW2 (Fig. 7A). Note that the SAL1 and SAL2 lipases are produced as pre-pro-enzymes (50). Signal peptidase I processes the pre-pro-lipase by cleaving the signal peptide (pre region) for secretion. Then, the secreted pro-lipase would be cleaved by an aureolysin to yield the mature lipase. However, cleavage of the pro-peptide has no effect on the lipase activity (60). Given this prior evidence, we also constructed an MW2 strain that could produce and export the MARS variant to the culture supernatant (exo-MARS strain) as a control. To do so, we fused the *mRFPmars* gene downstream of the *sal2* pre-pro region, which included the lipase signal peptide, replacing the gene region corresponding to the mature *sal2* lipase (Fig. 7A). In this manner, the mRFPmars protein would be exported following the same pathway as SAL2. Unfortunately, as occurred with other tagged LspU versions, the GFP tag impaired the LspU function. The LspU-GFP chimeric protein appeared to be associated with the cell envelope instead of being exported as the native LspU (Fig. S5), preventing us from studying LspU-GFP subcellular localization. Despite this, we first evaluated the functionality of mRFPmars fused proteins when expressing the native LspU. For this purpose, we transformed the SAL2-MARS and exo-MARS strains with pHRG-LspU and pHRG-LspU^STOP^ plasmids. Figure 7B shows that the expression of LspU reduced SAL2-MARS in the culture supernatant while increasing its presence in the SDS-soluble fractions from cell pellets, reproducing the effects observed with the SAL2 native protein. In contrast, the levels of chimeric exo-MARS protein in the supernatant were neither affected by LspU expression nor retained in cell pellets, indicating that the LspU effect was specific for SAL2-MARS (Fig. 7B). Moreover, this result also indicated that the SAL2 pro-peptide was not involved in the lipase trapping by LspU. Next, to control the chimeric SAL2-MARS and exo-MARS protein functionality, we measured red fluorescence and lipase activity in culture supernatants. Figure 7C and D show that both mRFPmars chimeras were functional, as red fluorescence and lipase activity could be detected in culture supernatants, and in agreement with the western blot results, LspU reduced lipase activity and red fluorescence in SAL2-MARS strains.

We then analyzed the subcellular location of SAL2-MARS. For this purpose, we inspected samples of overnight cultures of the SAL2-MARS and exo-MARS strains carrying the pHRG-LspU and pHRG-LspU^STOP^ plasmids under the fluorescence microscope. Unexpectedly, we found the presence of red aggregates of diverse sizes between cells of the SAL2-MARS strain constitutively expressing LspU (Fig. 7E). No red aggregates were observed either in exo-MARS strain or in the SAL2-MARS expressing the mutated *lspU* gene version, indicating that both mature lipase domains as well as LspU were required for their formation. Interestingly, these aggregates disappeared when cell fractions were treated with 1% SDS solution in comparison with those treated with PBS (Fig. 7F). Therefore, instead of being retained in the cell envelope as we first hypothesized, SAL2-MARS was being mostly accumulated in extracellular aggregates that pulled down together with bacteria during centrifugation. The fact that these aggregates were soluble in 1% SDS might explain the separation of SAL2-MARS from cell fractions. Interestingly, similar aggregates were observed in the wild-type strain expressing LspU, indicating that the formation of these aggregates was not a consequence of the chimeric SAL2-MARS protein (Fig. 7G).

### Amyloidogenic protein aggregates consisted of SAL2, PSMs, and LspU

**Fig 8:**
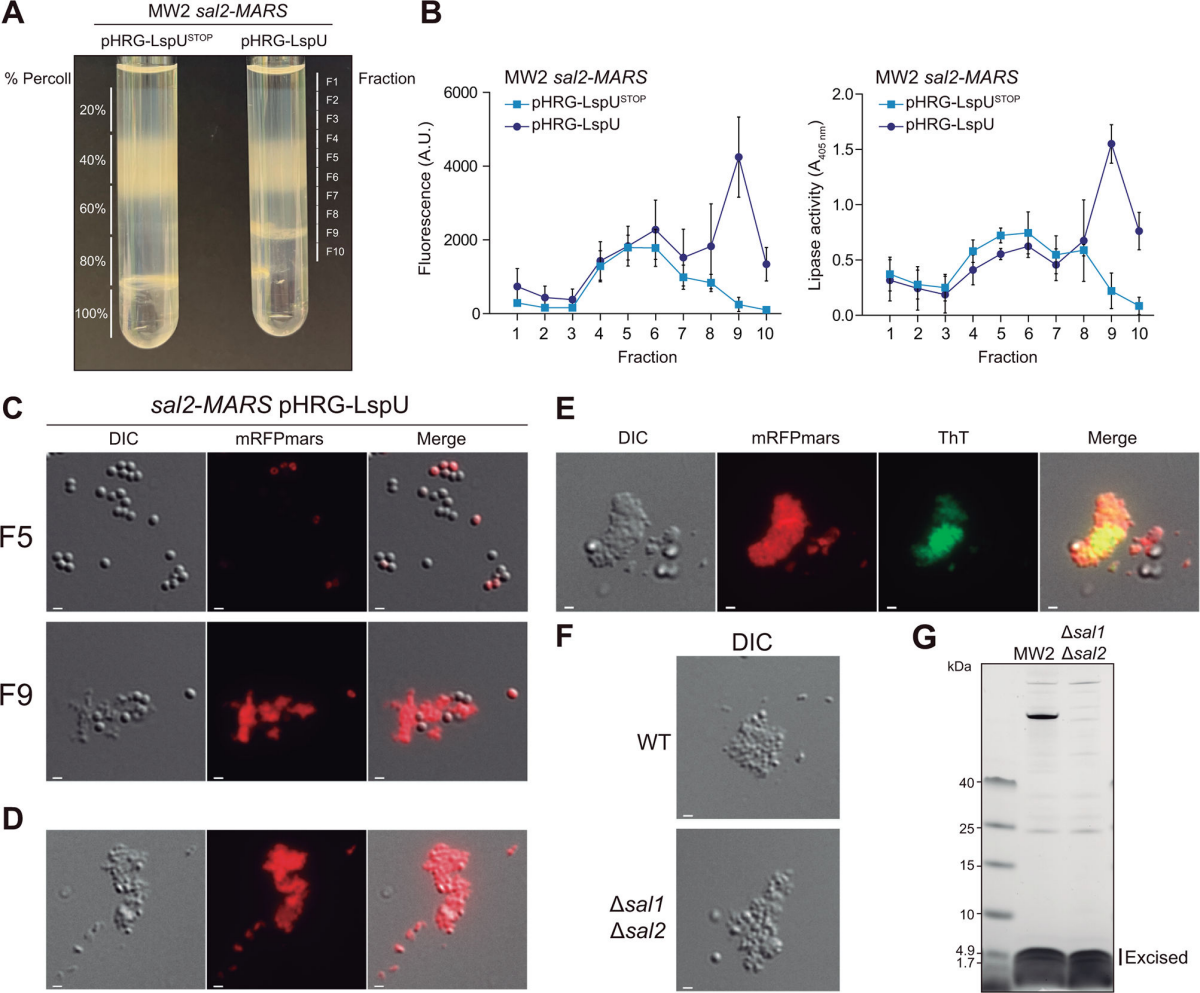
Protein aggregates are mainly composed of LspU, PSMs, and lipases. (**A**) A representative image of Percoll gradients performed with cell pellets from the MW2 SAL2-MARS strains carrying the pHRG-LspU and pHRG-LspU^STOP^ plasmids. Layers of different percentages of Percoll are indicated on the left and the extracted fractions (F1–F10) on the right. (**B**) Red fluorescence (A.U.) and lipase activity (*A*_405_) levels are plotted for each fraction extracted from the indicated strains. (**C**) Representative images of microscopic acquisitions in the DIC and red (mRFPmars) channels of the F5 and F9 fractions from the Percoll gradients of SAL2-MARS pHRG-LspU. Merged DIC and mRFPmars images (Merge) are shown. Scale bars, 1 µm. (**D**) Representative images of microscopic acquisitions in the DIC and mRFPmars channels of F9 after further sample centrifugation. (**E**) Representative images of microscopic acquisitions in the DIC, mRFPmars, and green (ThT) channels of purified aggregates from SAL2-MARS pHRG-LspU stained with ThT. Merged DIC, mRFPmars, and ThT images (Merge) are shown. (**F**) Representative images of microscopic acquisitions in the DIC channel of *S. aureus* MW2 (WT) and the Δ*sal1* Δ*sal2* strain. Scale bars, 1 µm. (**G**) Simple blue-stained SDS-PAGE separation of protein extracts of purified F9 fractions from the WT and Δ*sal1* Δ*sal2* strains after solubilization with 1% SDS. Protein bands excised for proteomic analysis are indicated.

According to our microscopy images, although the aggregates were apparently amorphous, a close detailed view of differential interference contrast images suggested that they could be formed by globular smaller units, which could be separated from the bacterial cells when grown in liquid cultures. Therefore, we aimed at purifying the LspU-mediated aggregates from bacterial cells based on differences in their density using Percoll gradients to analyze their conformation. To this end, we resuspended overnight cell cultures of the SAL2-MARS pHRG-LspU and pHRG-LspU^STOP^ strains in PBS and then applied a discontinuous Percoll gradient, comprising layers of 20, 40, 60, 80, and 100% (vol/vol) Percoll/PBS. We then centrifuged the tube gradients at 1,500 g for 5 min. The results showed that when constitutively expressing LspU, a distinctive natural fraction was formed between the 60% and 80% Percoll layers (Fig. 8A). To evaluate the layer in which SAL2-MARS aggregates could be retained, we divided the Percoll gradients into 300 µL fractions to measure red fluorescence and lipase activity (Fig. 8B). Fraction 9 (F9), which correlated with the differential fraction that appeared in LspU-expressing cultures (between the 60 and 80% Percoll layers), showed the highest red fluorescence and lipase activity levels when compared to the *lspU* mutated version (Fig. 8B). In addition, we visualized fractions 5 and 9 from the LspU-expressing cultures under the fluorescence microscope. F5 mainly contained bacterial cells, while F9 included red fluorescent aggregates and a fewer number of cells (Fig. 8C). To obtain a more purified version of F9, we further centrifuged the fraction for 5 min at 14,800 rpm and took the upper phase. Figure 8D shows that the number of bacterial cells was considerably reduced (Fig. 8D). We treated the purified aggregates with Proteinase K and DNase I as a control and visualized the results under a fluorescence microscope. Fig. S6 shows that Proteinase K digested the aggregates since no red fluorescence could be observed after the treatment, indicating that these aggregates were mainly composed of proteins. In contrast, the DNase treatment kept the aggregates mostly unchanged (Fig. S6). In addition, these protein aggregates were stained with thioflavin T (ThT). ThT fluorescence is a widely accepted indicator of amyloid fibril formation. Interestingly, as shown in Fig. 8E, the aggregates bound ThT, suggesting an amyloidogenic conformation (Fig. 8E). We applied the same Percoll gradient procedure to the LspU-expressing MW2 strain, and similar aggregates could also be observed (Fig. 8F).

Since lipases are prone to aggregation due to the presence of hydrophobic surfaces (65–67), we evaluated whether the formation of protein aggregates required the presence of lipases. Percoll gradients were performed with overnight cultures of the MW2 Δ*sal1* Δ*sal2* pHRG-LspU strain, and their aggregates were purified. Figure 8F shows that aggregates were also formed in the absence of lipases, indicating that the constitutive expression of LspU was responsible for protein aggregate formation.

To analyze protein composition of the aggregates, we loaded the corresponding fractions in 10–20% gradient Tris-Tricine polyacrylamide gels after their solubilization in 1% SDS. Fig. 8G shows that aggregate fractions were mainly composed of three major proteins in the case of the MW2 wild-type strain, whose sizes might correspond to pro-lipases (~70 kDa), LspU, and an unknown small protein running close to LspU (about and below the 4.9 kDa mark; Fig. 8G). In the case of the MW2 Δ*sal1* Δ*sal2* mutant, the main bands were attributed to both small proteins. To further confirm this, we excised the small protein bands from the gel and performed LC-MS/MS for protein identification in the Proteomic Services of CNB-CSIC. After filtering the data by score and protein size, the mass spectrometry analysis identified that trypsin-digested peptides from excised bands corresponded to PSMβ1, PSMα1, PSMα4, δ-toxin, and LspU (Table S2). Altogether, these results showed that the protein aggregates induced by LspU mainly consisted of LspU, PSMs, and lipase proteins, which might adopt an amyloidogenic configuration.

### LspU shared PSMs transporter systems

Finding some PSMs as a part of the LspU-mediated amyloidogenic protein aggregates suggested a close relationship between these small proteins. The α-type PSMs require specialized export systems to cross through cell membranes after their production, such as the Pmt and AbcA transporter systems (68, 69). Considering the predicted structural similarities between LspU and PSMs, we wondered whether LspU might also be exported through these transporter systems. To prove this, we used the combinatorial mutants that lacked all *psm* genes as well as the *pmt* and *abcA* transporter genes, constructed in the *S. aureus* USA300 LAC strain by M. Otto’s group. The deletion of these transporter systems alone cannot be performed because PSM accumulation inside cells affects bacterial survival (68). Since the *S. aureus* LAC derivative mutants were resistant to erythromycin, we generated the pHRT-LspU^FLAG^ plasmid that included tetracycline as an antibiotic marker instead of erythromycin to express the FLAG-tagged LspU in these strains. Our western blot results showed that in the absence of Pmt and AbcA transporters, LspU^FLAG^ was retained in the cell pellet fractions and could not be extracted by 1% SDS (Fig. S7). This result indicated that LspU shares PSMs transporter systems and provided additional evidence to support LspU as a novel PSMα

### LspU trapped lipases in cell fractions and biofilm structures in the absence of PSMs

**Fig 9:**
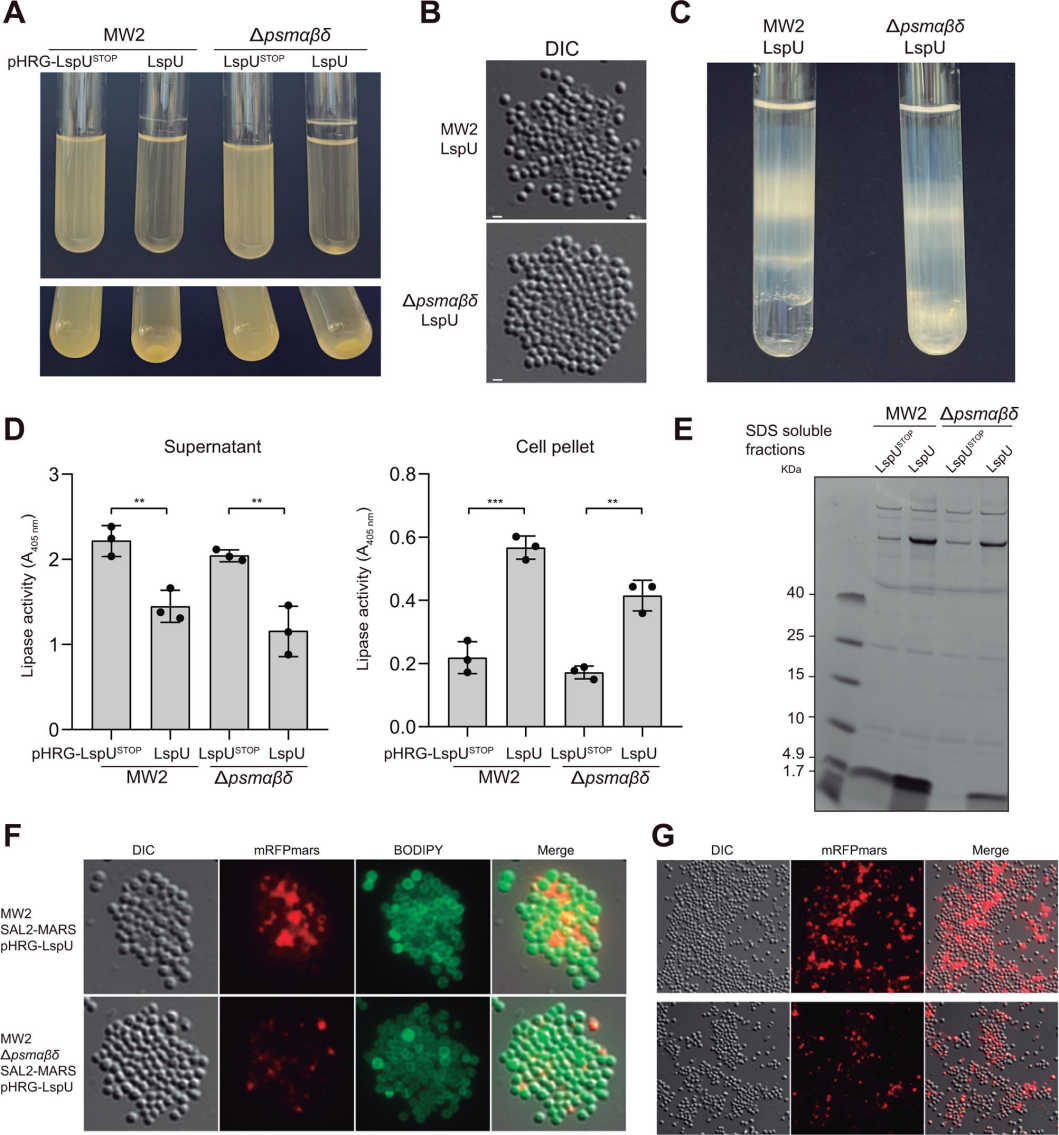
SAL2-MARS is retained in cell surface and biofilm structures in the absence of PSMs. (**A**) Phenotypic comparison of the *S. aureus* MW2 and Δ*psm*αβδ mutant strains carrying the pHRG-LspU and pHRG-LspU^STOP^ plasmids. Biofilm formation and bacterial aggregation phenotypes were observed after incubation for 16 h at 200 rpm and 37°C in glass tubes containing 5 mL of MH broth. (**B**) Representative images of microscopic acquisitions in the DIC channel of *S. aureus* MW2 and Δ*psm*αβδ carrying the pHRG-LspU plasmid. Scale bars, 1 µm. (**C**) A representative image of Percoll gradients performed with cell pellets from *S. aureus* MW2 and Δ*psm*αβδ carrying the pHRG-LspU plasmid. (**D**) Bar plots showing the lipase activity in supernatants and cell pellet fractions from MW2 and Δ*psm*αβδ carrying the pHRG-LspU and pHRG-LspU^STOP^ plasmids. The lipase activity was assayed on OD_600_ normalized spent culture supernatants or protein concentration normalized cell pellets harvested by centrifugation after growth in MH broth for 16 h at 37°C and 200 rpm. The release of p-nitrophenol from p-nitrophenyl butyrate was measured by spectrophotometry at 405 nm. Asterisks represent statistical significance (**, *P* < 0.005; ***, *P* < 0.0005; *t*-test). (**E**) Simple blue-stained SDS-PAGE separation of protein extracts of the cell pellet fractions after solubilization with 1% SDS. (**F**) Representative images of microscopic acquisitions in the DIC, red (mRFPmars), and green (BODIPY FL Vancomycin) channels of SAL2-MARS WT and Δ*psm*αβδ strains carrying pHRG-LspU. Merged DIC, mRFPmars, and BODIPY images (Merge) are shown. (**G**) Representative images of microscopic acquisitions in the DIC and mRFPmars channels of samples taken from the biofilm ring formed over air-liquid interface in glass tubes.

PSMs can aggregate to form amyloid-like structures (70–73). To investigate whether PSMs were required for the protein aggregate formation, we generated a Δ*psm*αβδ mutant in the MW2 strain by deleting the two loci that included the *psm*α*1-4* and *psm*β*1-2* operons and mutating δ-toxin first ATG. We then complemented the resulting MW2 Δ*psm*αβδ mutant strain with the pHRG-LspU and pHRG-LspU^STOP^ plasmids and evaluated the phenotypes of the transformants. Interestingly, the Δ*psmα*βδ mutant strain showed a thicker biofilm ring in glass tubes than the WT strain when LspU was expressed (Fig. 9A). In contrast, visualization under the microscope of bacterial samples from 16 h liquid cultures revealed the absence of protein aggregates in the Δ*psm*αβδ pHRG-LspU strain, while bigger cell clusters were found in comparison to the WT pHRG-LspU strain (Fig. 9B). Also, the Percoll gradient results showed that protein aggregates could not be purified from the Δ*psm*αβδ pHRG-LspU strain (Fig. 9C). This suggested that the biofilm ring phenotype was unrelated to the formation of protein aggregates.

The fact that the big extracellular aggregates could not be formed in the Δ*psm*αβδ pHRG-LspU strain posed another question related to the actual subcellular localization of lipases in the absence or reduced expression of PSMs. To investigate this, we first compared lipase activities of supernatant and cell pellet fractions from MW2 WT and Δ*psm*αβδ mutant strains expressing LspU. Figure 9D shows that lipase activity of WT and Δ*psm*αβδ pHRG-LspU strains decreased in supernatants and increased in cell pellet fractions in comparison to the corresponding control pHRG-LspU^STOP^ strains, indicating that LspU was still inducing lipase trapping to cell pellets, even in the absence of PSMs. We confirmed this by protein gel staining of cell pellet fractions treated with SDS, which showed higher levels of protein bands corresponding to lipases and LspU in the Δ*psm*αβδ strain when LspU was expressed (Fig. 9E). Moreover, protein gels also showed that the second small protein band, running below the 4.9 kDa mark and above LspU, disappeared in the Δ*psm*αβδ mutant, confirming that it corresponded to PSMs.

To explore the subcellular location of lipases when LspU was expressed in the absence of PSMs, we generated a Δ*psm*αβδ strain that carried the chimeric SAL2-MARS protein through allelic replacement. We complemented the resulting strain with the pHRG-LspU and pHRG-LspU^STOP^ plasmids and grew the transformants in liquid cultures for 16 h. We then prepared bacterial samples, which were stained with BODIPY FL Vancomycin to label their cell surfaces and visualized under the fluorescence microscope. Figure 9F shows that SAL2-MARS localized between cells, but as smaller red clumps when LspU was expressed. This suggested that LspU also contributed to retaining SAL2-MARS around bacterial cells in the absence of PSMs (Fig. 9F). We also analyzed the biofilm ring formed in glass tubes, which we collected and visualized in a similar manner. Images showed that SAL2-MARS was associated with biofilm structures even when PSMs were lacking. However, in this case, the protein aggregates were not as dense as those from the WT strain (Fig. 9G). Altogether, these results indicated that LspU induced lipase trapping at cell surfaces and biofilm structures regardless of the presence of PSMs, although PSMs made more compact biofilm structures in conditions in which LspU was constitutively expressed.

## DISCUSSION

The “dark matter” of bacterial proteomes is made of hidden small proteins with unknown functions that are waiting to be found. Novel discoveries of small proteins in bacteria are often restricted by culture conditions, strain diversity, and technological limitations. Therefore, comprehensive complementary analyses are required to maximize small protein detection (1, 8). In this work, we have updated the repertoire of small proteins in *S. aureus* (Table S1) by analyzing a combination of in-house and public multiomic data sets, thus complementing previous studies from this pathogen (19, 21, 22). The comparison of SaSP sequences shorter than 50 aa in length (SaSPs < 50) showed that only 18 and 21 out of the 90 SaSPs identified in our analysis correlated with small proteins listed in Fuchs et al*.* and Durrant et al*.* studies, respectively (21, 22) (Table S1). All the novel SaSPs were found by Ribo-seq analysis, indicating that this technique was more sensitive for small protein detection than shotgun proteomics. All the in-house and publicly available data analyzed in this study can be browsed in a web server available at https://rnamaps.unavarra.es/. Beyond the identification of small proteins, this information might be useful for determining the transcript and ORF boundaries of any expressed gene as well as evaluating its translation.

Among the novel SaSPs identified in our multiomic analysis, we were interested in small proteins that were genetically associated with important virulence genes, which could indicate their putative participation in the *S. aureus* pathogenesis process. The *sal1* mRNA, which produces the extracellular lipase SAL1, was particularly motivating since it encodes two SaSPs of 21 aa, LspU, and LspD at the former 5′-UTR and 3′-UTR, respectively (Fig. 2). Lipase activity is crucial for *S. aureus* infections (74), so the discovery of small proteins that were co-translated with SAL1 suggested an unknown functional correlation.

Previous studies showed how the expression of *sal1* was significantly downregulated in a rabbit skin infection model (75) while being upregulated in both a murine bone infection model and a prosthetic joint infection of an adult male patient (76, 77). This could mean that LspU, SAL1, and LspD are produced during infections of deep tissues but may not be dominant in superficial infections, such as the skin.

Our sequence and structural analyses indicated that LspU and LspD could be considered as novel members of the α-class PSMs, both showing similar features to PSMα1-4: highly conserved sequences among *Staphylococcus* species and an amphipathic helix with hydrophobic and hydrophilic/charged residues situated on opposite faces (Fig. 2).

Interestingly, PSMα1-4 showed similar expression patterns to those of the *sal1* mRNA (76), which included LspU and LspD, indicating that a transcriptional correlation might have been evolutionarily selected, supporting putative functional relationships between these SaSPs and lipases.

In this study, we focused on the uORF LspU as an intriguing example for further characterization. First, considering that lipase-encoding genes are tightly regulated, we thought that LspU could be a uORF working as a *cis*-acting post-transcriptional element modulating SAL1 expression. Note that our multiomic analysis revealed nine SaSPs encoded by former 5′-UTRs, which could be considered as putative *cis*-acting regulatory elements, according to their genomic position relative to the main ORFs. In fact, one of them corresponded to a previously identified 26-aa leucine-rich small protein (SaSP_069 in Table S1), which worked as an attenuator peptide repressing the branched-chain amino acid synthesis *in S. aureus* (31). Since LspU has a similar size to SaSP_069 and was encoded at a reasonable distance from the *sal1* ORF, we investigated whether the LspU uORF could participate in modulating the production of its associated SAL1 ORF, as previously described (5, 31). Although analysis of the putative secondary RNA structures of the 5′ *sal1* mRNA region indicated RNA conformations that could work as a *cis*-acting regulatory element (data not shown), our translational reporter experiments showed that the production of SAL1 was not affected when LspU uORF was mutated, at least in the tested conditions (Fig. S3). Further investigations will be required to evaluate whether translation of LspU might modulate SAL1 expression.

Second, we looked for phenotypes that could be associated with lipase activity when LspU was expressed. Our results showed that the constitutive expression of LspU reduced the lipase activity from the culture supernatant (Fig. 3C), a consequence of the reduction of both SAL1 and SAL2 protein levels from this protein fraction (Fig. 4). This drop in lipase abundance was explained by the retention of SAL1 and SAL2 protein in the cell pellet fractions (Fig. 5), which suggested that LspU could be promoting lipase interaction with cellular fractions. Although the results showed that this hypothesis was true, understanding how LspU expression led to the observed phenotypes was more complicated than anticipated. When investigating the localization of fluorescent chimeric SAL2-MARS protein when LspU was expressed, the visualization of cell pellets under the fluorescence microscope showed the formation of SDS-soluble protein aggregates that pulled down together with the cells during centrifugation (Fig. 7). Interestingly, the protein aggregates could be purified by Percoll gradients (Fig. 8), and the composition analyses revealed that they were mainly composed of PSMs, LspU, and lipases (Fig. 9). These results suggested that LspU could work as a nucleator agent for PSMs and SAL proteins. Note that the expression of PSMs and lipases is growth phase dependent and strongly upregulated by the Agr system (56, 78, 79). In such conditions, PSMs are produced in extremely high amounts (56, 68), and activation of *sal1* mRNA expression (here artificially performed by the constitutive expression exerted by P_hyper_ promoter) could promote the formation of protein aggregates that trap lipases close to bacterial cells in biofilm structures.

Several studies have confirmed that PSMs can aggregate to form amyloid-like structures, which could contribute to biofilm structures (70–73). For example, the δ-toxin was responsible for the formation of protein aggregates in planktonic cultures of *S. aureus* (80, 81), which bind ThT as well as the protein aggregates formed by LspU expression (Fig. 8). It has been proposed that, according to the N-terminus formylated state of the δ-toxin, different protein aggregates can be formed. Thus, formylated δ-toxin and deformylated δ-toxin tend to form fibrils and oligomers, respectively (80). Oligomers were formed by additional PSMs, including PSMα1-4. Similar to the LspU-mediated aggregates, which were also oligomers including PSMs, LspU, and lipases, the fibrils promoted by δ-toxin could be disrupted by SDS treatments (81). Interestingly, the deletion of the PSMs-encoding genes reduced the formation of protein aggregates, but LspU and lipases were still associated with the cell surface. This association was disrupted by the addition of 1% SDS. Microscopy analyses revealed that SAL2-MARS was located on spots on the cell surface (Fig. 9), indicating that LspU and lipases were still interacting in the absence of PSMs. This seemed logical considering that *lspU* and *sal1* were co-transcribed, and they should be functionally related, validating the hypothesis of lipases interacting with cellular fractions through LspU.

Based on the whole results presented here, we propose that, under the appropriate conditions, LspU could be trapping the lipases by confining them to an area of influence of the bacterial cells. This would be helpful considering that bacteria commonly live in nature attached to different kinds of biotic and abiotic surfaces, establishing microcolonies or biofilms. As a result, when microcolonies are under hydrodynamic conditions, extracellular compounds produced by bacteria could diffuse away from the microcolony. In the case of *S. aureus* extracellular lipases, which are heavily produced, their diffusion could be unproductive by preventing bacteria from using their by-products. It would seem that *S. aureus* has solved this problem by co-transcribing SAL1 with LspU as a “retention” factor. Moreover, the expression of LspU also increased *in vitro* biofilm formation (here in the form of a ring in the glass tubes). Probably, the generation of protein aggregates reduced PSMs functionalities by inhibiting their surfactant activity. This correlated with our previous studies showing that the absence of PSMs leads to biofilms with enhanced thickness (Fig. 9A) (82, 83). It is noteworthy that this process seems reversible since protein aggregates could be disassembled in certain conditions (here artificially produced by the action of SDS). A reversible phenomenon could provide bacterial adaptation during environmental changing conditions. It is feasible to propose that when disassembly of LspU-mediated aggregates occurs, PSMs could be liberated for the restructuration of biofilms and/or promoting host cell lysis at the infection sites, allowing dissemination of bacteria and/or generating free lipids to be processed by lipases for *S. aureus* benefit. Although it is clear that further *in vivo* experiments are required to demonstrate the implications of LspU during *S. aureus* infections, we can anticipate that LspU would play a relevant role. Besides, further characterization of the LspU functions in combination with LspD will help confirm these small proteins as novel PSM members, increasing the relevance of this peculiar small protein family and opening new questions about their biological relevance in *S. aureus* pathogenesis and the relationship between PSMs and lipases.

## MATERIALS AND METHODS

### Strains, plasmids, oligonucleotides, and growth conditions

The bacterial strains, plasmids, and oligonucleotides used in this study are listed in Tables S3 to S5 . *S. aureus* and *Escherichia coli* strains were grown in MH broth (Sigma-Aldrich) and Luria-Bertani (LB) broth (Pronadisa), respectively. The B2 (casein hydrolysate, 10 g L^−1^; yeast extract, 25 g L^−1^; NaCl, 25 g L^−1^; K_2_HPO_4_, 1 g L^−1^; glucose, 5 g L^−1^; pH 7.5) and SuperBroth (tryptone, 30 g L^−1^; yeast extract, 20 g L^−1^; MOPS, 10 g L^−1^; pH 7) media were used to prepare *S. aureus* and *E. coli* competent cells, respectively. For selective growth, media were supplemented with the appropriate antibiotics at the following concentrations: erythromycin, 10 µg mL^−1^; ampicillin, 100 µg mL^−1^; chloramphenicol, 10 µg mL^−1^; and tetracycline, 5 µg mL^−1^.

### Ribosome profiling and RNA sequencing

Preinocula cultures of *S. aureus* 15981 strain were grown in 10 mL MH at 37°C and 180 rpm overnight. Preinocula cultures were diluted to a final OD_600_ of 0.05 in 2 L-Erlenmeyer flasks containing 400 mL of MH broth, which were incubated with agitation (200 rpm) at 22°C and 37°C until cells reached an early exponential phase (OD_600_ = 0.4).

For total RNA sequencing, we recovered 20 mL of cultures to extract total RNAs as previously described (84). Total RNAs were sequenced at the Gene Expression Analysis Platform from IBPM-CNRS, and fastq files were aligned to the *S. aureus* NCTC 8325 reference genome (NC_007795.1) using SeqMan NGen software from DNASTAR package.

For ribosome profiling, 2 min before harvesting, chloramphenicol (1 mM) was added to the cultures. Cell cultures were harvested by centrifugation for 10 min at 4,400 *g* and 4°C. Bacterial pellets were resuspended in 500 µL of lysis buffer R (20 mM Tris-HCl pH 8.0, 100 mM NH_4_Cl, 10 mM MgCl_2_, 0.1% Nonidet p-40, 0.4% Triton X-100, 1 mM chloramphenicol, and 100 U mL^−1^ DNase I) and lysed twice in Lysing Matrix B tubes for 40 s at 6.0 m s^−1^ in a FastPrep-24 instrument (MP Biomedicals). Tubes were centrifuged for 15 min at 21,000 × *g* and 4°C. Supernatants were recovered in 1.5 mL Eppendorf tubes, and RNA concentrations were determined. To obtain ribosome footprints, 1,000 U of Micrococcal Nuclease S7 (Roche) was added per 1 mg of RNA. Samples were incubated for 1 h at room temperature with rotation at 190 rpm. The reaction was quenched by the addition of EDTA to a final concentration of 6 mM. A fraction of RNA samples was loaded onto the linear sucrose gradients 5%–50% prepared on the Gradient Master IP (Biocomp). Tubes were centrifuged at 260,000 × *g* (39,000 rpm, SW41 Ti rotor, Beckman Coulter) for 3 h at 4°C using a piston gradient fractionator (Biocomp) combined with a fraction collector (Gilson). Fractions containing 70S and 70S with protected mRNA were stored at −20°C until needed.

Hot acid phenol-chloroform RNA extraction from monosomes and library preparations for RNA sequencing were carried out as previously described (85). Raw fastq sequencing data were trimmed using cutadapt to remove Illumina adapters (86). To remove rRNA sequences, trimmed fastq files were aligned to *S. aureus* ribosomal sequences using Bowtie v.1.2.2 (87). The rRNA-filtered fastq files were then aligned to the *S. aureus* NCTC 8325 reference genome (NC_007795.1) using Bowtie v.1.2.2. The obtained SAM files were sequentially converted to BAM, bedgraph, and BigWig files using samtools v1.9, stranded-coverage, and bedGraphToBigWig programs, respectively (88).

### Shotgun proteomics

Preinocula cultures of *S. aureus* 15981 strain were grown in 5 mL MH at 37°C and 200 rpm overnight. Preinocula cultures were diluted to a final OD_600_ of 0.05 in 500-mL Erlenmeyer flasks containing 100 mL of MH broth and incubated at 22°C and 37°C with agitation (200 rpm) until cells reached an early exponential phase (OD_600_ = 0.4). Then, cell cultures were divided into two 50 mL Falcon tubes and harvested by centrifugation for 10 min at 4,400 × g and 4°C and sample fractionation proceeded as follows.

For total protein fractions, pelleted cells from one of the 50 mL Falcon tubes were washed with PBS and resuspended in 1 mL of proteomic buffer. Proteomic buffer was freshly prepared by mixing equal amounts of buffer A (10% SDS), buffer B (300 mM triethylammonium bicarbonate, 15 mM Tris-[carboxyethyl]phosphine), and buffer C (30 mM chloroacetamide). Samples were transferred to Lysing Matrix B tubes containing glass beads, and bacteria were mechanically lysed using FastPrep apparatus at 6.0 m/s twice. Samples were then centrifuged at 21,000 *g* for 10 min, and supernatants were collected and incubated at 60°C for 30 min. Samples were stored at −80°C until needed.

For membrane protein fractions, the remaining pelleted cells from the other 50 mL Falcon tube were resuspended in 970 µL of lysis buffer (MgCl_2_ 10 mM, CaCl_2_ 1 mM, and Tris 50 mM), 20 µL of lysostaphin, and 10 µL of phenylmethylsulfonyl fluoride (PMSF) and incubated for 30 min at 37°C. Then, the resuspension was lysed twice in Lysing Matrix B tubes using FastPrep (6.0 m s^−1^) and centrifuged at 21,000 × *g* for 10 min. The supernatant was ultracentrifuged at 100,000 g for 1 h at 4°C, and the pellet, which corresponded to the membrane fraction, was resuspended in 50 µL of proteomic buffer. Samples were incubated at 60°C for 30 min and stored at −80°C until needed.

For the extracellular protein fractions, proteins from the supernatants were precipitated using trichloroacetic acid (TCA). Culture supernatants were filtered through a 0.22 µm sterile pore filter. Fifteen percent TCA was added, and samples were incubated for 1 h on ice. Precipitated proteins were recovered by centrifugation at 11,000 rpm for 30 min. The precipitated extracellular proteins were washed with 1 mL of cold acetone and resuspended in 50 µL of proteomic buffer. The samples were incubated at 60°C for 30 min and stored at −80°C prior to proteomic processing.

Protein fractions were processed at the Proteomic Facility from CNB-CSIC. Protein samples were tryptically digested using S-Trap filter (Protifi, Huntington, New York) according to the manufacturer’s protocol. Digested samples were individually analyzed by nano-Liquid Chromatography coupled to Electrospray Ionization Tandem Mass Spectrometry (nanoLC-ESI-MS/MS) analysis using an Ultimate 3000 nano high-performance liquid chromatography (HPLC) system (Thermo Fisher Scientific) coupled online to an Orbitrap Exploris 240 mass spectrometer (Thermo Fisher Scientific). According to the sample complexity, a 60 min gradient was applied. The obtained MS/MS raw data were analyzed using MaxQuant software (https://www.maxquant.org). To identify digested peptides, we used a custom database including all putative proteins larger than 10 amino acids translated from the *S. aureus* NCTC 8325 genome (NC_007795.1) in all six open reading frames using the OrfM software (89). Positions of identified peptides were mapped to genomic coordinates (excluding overlapping annotations) and correlated to their corresponding intensities to generate bedGraph files, which were converted to BigWig files using bedGraphToBigWig. The generated peptide intensity wig files were loaded into JBrowse.

### Combination of multiomics data in JBrowse

Genome-wide data from RNA-seq, Ribo-seq, and shotgun proteomics analyses were compiled into a web server publicly available at http://rnamaps.unavarra.es, which is based on the JBrowse, a JavaScript-based genome browser (39), which allows graphical visualization of the compiled data throughout the genomic coordinates. We used *S. aureus* NCTC 8325 fasta file (NC_007795.1) as the genome reference. The *S. aureus* NCTC 8325 .gff files from NCBI and PATRIC databases were included as annotation tracks. An additional annotation track was created by reannotating this genome sequence using the Bakta Web application (42). In addition to the Ribo-seq, RNA-seq, and shotgun proteomic tracks generated in this study, the browser was complemented with tracks including data from previous studies that were reanalyzed using the *S. aureus* NCTC 8325 genomic sequence as a reference. The public genome-wide data included in JBrowse corresponded to the following studies: Long RNA-seq sequencing of wild-type *S. aureus* 15981 strain (SRA accession SRR397556) (43), TSS mapping performed in *S. aureus* Homeland Sc01 strain (SRA accession SRS468014) (44), Ribo-seq analysis in *S. aureus* JE2 strain grown in TSB and minimal media (replicates 1 and 2, PRJNA299306) (45), and shotgun proteomic analysis of extracellular vesicles purified from *S. aureus* CICC 10384 strain (PRIDE accession number PXD034259) (46).

### Identification of small proteins

To identify small proteins, the omic data loaded in the JBrowse application were correlated with a custom file containing all six open reading frames smaller than 50 amino acids and their corresponding upstream regions to look for putative RBSs. This file was filtered using the consensus RBS region sequence, AGGAGG(N)_5-9_[A,T,G]TG, start codons ATG, TTG, and GTG and allowing up to two mismatches in the RBS sequences. Then, the mapping of the Ribo-seq signals (number of reads) at the RBS regions and the proteomic signals (number of identified peptides) at the sORFs was quantified. The proteomic analysis detected only one protein smaller than 50 amino acids, indicating that it was not sensitive enough for small protein detection. Therefore, we continued the identification of small proteins using the Ribo-seq signals and considering a threshold of five reads in at least one biological replicate. Finally, the data were manually curated by visualizing track signals in JBrowse. We excluded sORF candidates that were assigned with (i) a weak RBS, (ii) sORFs included in repeated regions, and (iii) erroneous signal assignation due to the translation of adjacent ORFs, wrong gene annotations, or genomic differences between *S. aureus* 15981 and the reference strains. As a result, only small proteins showing logical peak signals covering the RBS region were included in Table S1.

### Small protein sequence comparative analysis

To look for orthologs of LspU and LspD small proteins in bacterial species, we performed tblastn analysis (https://blast.ncbi.nlm.nih.gov/). To maximize the detection of the orthologous sORFs and considering that the protein query sequences were short, the tblastn algorithm parameters were modified by setting the expected threshold to 100, the “max target sequences” to 5,000; and *S. aureus* genomes excluded from the search set. Once an ortholog was detected in a particular species, a representative genome was downloaded to extract the complete sORF sequences. sORF sequences were compiled in Geneious Prime software to perform comparative sequence analyses.

### Plasmid constructions

Plasmids used in this study are listed in Table S4 and were engineered as previously described (26). Briefly, PCR fragments were amplified from chromosomic or plasmidic DNA with the DreamTaq DNA polymerase, Phusion High-Fidelity DNA Polymerase (Thermo Scientific) or Kapa Polymerase (Roche), using the oligonucleotides listed in Table S5. The PCR products were run and purified from agarose gels using NZYGelpure kit (NZYtech) and ligated into the pJET 1.2 vector (Thermo Scientific). The resulting plasmids were used to transform *E. coli* IM01B cells. Plasmids were purified from overnight cultures with the NZYMiniprep kit (NZYTech) and verified by Sanger sequencing. When required, DNA fragments were excised using FastDigest restriction enzymes (Thermo Scientific) and ligated into the appropriate vector with the Rapid DNA ligation kit (Thermo Scientific). The final plasmids were introduced into *S. aureus* strains by electroporation, as previously described (90). Detailed procedures for the construction of the plasmids used in this study are included in the supplemental material.

### Chromosomal mutagenesis

For the construction of the chromosomal mutants generated in this study (Table S3), a two-step homologous recombination based on the pMAD plasmid (61) was used, as previously described (38). The resulting mutant strains were verified by PCR using the corresponding flanking oligonucleotides (Table S5) and Sanger sequencing. For PCR verification of the Δ*sal1* and Δ*sal2* mutants, oligonucleotides 376 and 377, and 382 and 383 were used, respectively. For the verification of *sal1^6×His^* and *sal2^6×His^* mutations, oligonucleotide pairs 376/442 and 383/442 were used, respectively. Checking of P*_sal2_::sal1^6×His^* Δ*sal1*was performed using oligonucleotides 382 and 383. Oligonucleotides 382 and 575 were used to check for *sal2-MARS* and exo-*MARS* mutations. For *psm*α and *psm*β operon mutants and the *psm*δ, oligonucleotide pairs 639/644, 645/650, and 651/656 were used, respectively. Additionally, for checking the *psm*δ mutant, the PCR product was digested with MunI restriction enzyme, since the corresponding recognition site was introduced when generating the mutation.

### Evaluation of small protein expression

DNA regions including the RBS and the first three codons of the corresponding small proteins were fused to an ATG-less *gfp* gene to generate translational fluorescent reporter plasmids. Plasmids were electroporated into *S. aureus* 15981 strains, and GFP levels were determined. Overnight cultures of *S. aureus* 15981 strains carrying the plasmids corresponding to the selected SaSPs grown in LB were diluted 1/40 in 96-well plates in a final volume of 200 µL. After 4 h of incubation at 37°C with agitation, OD_600_ and GFP fluorescence intensity were measured using a BioTek Synergy H1 microplate reader. The fluorescence background obtained from the *sbrB* mutant carrying a premature stop codon (*S. aureus* 15981 pTL81_STOP^34^, background control strain) was subtracted from all fluorescence reads.

### Growth curves

Overnight cultures were initially inoculated at an OD_600_ of 0.025 in 250 mL Erlenmeyer flasks containing 50 mL of MH broth supplemented with the corresponding antibiotic. Optical density (OD_600_) reads were performed every 1 hour.

### Fractionation of bacterial cultures

Cultures were inoculated to an initial OD_600_ of 0.05 in 20 mL of MH broth supplemented with the corresponding antibiotics. Then they were harvested by centrifugation for 10 min at 4,400 × *g* and 4°C when they reached the indicated growth phase. For the extraction of whole cell protein extracts, the resulting cell pellets were resuspended in PBS and transferred to Fast-Prep tubes containing acid-washed 100 µm glass beads (Sigma). Then, bacterial suspensions were lysed using the FastPrep-24 instrument (MP Biomedicals) twice, for 45 s and at a speed of 6 m s^−1^. Whole cell extracts were obtained after centrifugation (21,000 × *g*, 4°C) for 10 min. When needed, protein concentration was quantified using the Bradford protein assay (Bio-Rad), and samples were prepared at the desired concentration.

For the obtention of the extracellular fractions, overnight cultures were centrifuged as described before. The resulting supernatants were filtered through 0.22 µm sterile pore filters and stored at −20°C until needed. SDS-soluble fractions were extracted, with slight modifications, as previously described (62). Briefly, bacterial pellets from the cultures, normalized by OD_600_, were incubated at 37°C for 10 min with 100× Halt Protease Inhibitor (Fisher Scientific), followed by a 30 min incubation with 1% (wt/vol) SDS and centrifuged. The resulting supernatant corresponded to the SDS-soluble fraction. The remaining pellets were then treated to extract the soluble proteins, and proteins of those fractions were extracted as previously described and stored at −20°C until needed.

### Protein gel staining and western blotting

Sample fractions were prepared in 6× Laemmli buffer and boiled at 95°C for 5 min. Samples were separated onto Stain-free 12% SDS-Tris-glycine polyacrylamide gels (BioRad), and proteins were visualized. When other acrylamide percentage gels were used for protein visualization, the separated proteins were stained with SimplyBlue SafeStain (Invitrogen). For western blot analysis, resolved proteins were transferred to nitrocellulose membranes (Amersham Biosciences), blocked in PBS containing 0.1% Tween 20 and 5% milk, and incubated with the corresponding antibodies. For low molecular weight proteins, Invitrogen Novex Tricine Mini Protein Gels 10%–20% (Invitrogen) were used. For staining them, SimplyBlue SafeStain (Invitrogen) was used, and for western blot, gels were transferred to ethanol-activated 0.2 µm pore size polyvinylidene difluoride (PVDF) membranes (Fisher Scientific). The antibodies used in this study were anti-FLAG-HRP antibody (Sigma, 1:1,000), anti-His-HRP (Sigma, 1:2,000), mouse anti-GFP antibody (Living Colors, Clontech, 1:5,000), and peroxidase-conjugated goat anti-mouse immunoglobulin G and M antibodies (Pierce-Thermo Scientific, 1:2,500). Membranes were then developed with ECL Prime Western Blotting Detection Reagent (Cytiva Amersham) using a ChemiDoc Imaging System.

### Lipase activity

Lipase activity in culture supernatants was measured in 1 or 5 µL of samples, normalized to OD_600_ of 3 for *S. aureus* MW2 and 15981 strains, respectively. Lipase activity in protein extracts was measured in 5 µL of samples normalized to 0.2 µg µL^−1^ of total protein extracts. Sample aliquots were mixed with lipase buffer, composed of p-nitrophenyl butyrate (Sigma) diluted in a succinate buffer solution (pH 6.5, 0.2 mM CaCl_2_, and 1% Triton X-100) in a final volume of 200 µL. Samples were incubated at 37°C for 1 h, and absorbance at 405 nm was measured with the BioTek Synergy H1 microplate reader.

### Fluorescence microscopy imaging

For visualization of *S. aureus* strains, gradient fractions, aggregates, and biofilm rings, samples were placed on glass slides and covered with coverslips. When indicated, samples were treated with BODIPY FL vancomycin (Invitrogen) at 1 µg/mL and incubated for 30 min. Samples were directly visualized using an HCX PL APO 100×/1.40–0.70 oil objective in a Leica DMi8 fluorescence microscope controlled by LAS X software.

### Percoll gradient

For the separation of the culture based on the density of its components, gradients using Percoll (Sigma) were performed. Briefly, 15 mL of the overnight cultures was centrifuged, and the corresponding pellets resuspended in 2 mL of 1× PBS. A total of 300 µL was then centrifuged at 1,500 × *g* for 5 min in the Percoll density gradient columns made up of 20, 40, 60, 80, and 100% Percoll in PBS.

For the isolation of the culture aggregates, the 80% Percoll fraction phase was collected in Eppendorf tubes. After a 5 min centrifugation at 21,000 × *g*, the upper phase, which corresponded mainly to the aggregates, was taken and placed in another Eppendorf tube containing PBS. After a 5 min centrifugation at 21,000 × *g*, the resulting pellet was washed again with PBS and centrifuged at 21,000 × *g* for 5 min. Finally, the pellets, which corresponded to the aggregates fraction, were resuspended in PBS for being directly visualized in the microscope or their proteins solubilized in 1% SDS, as previously described.

### Characterization of biofilm and aggregates composition

Five milliliter overnight cultures of *S. aureus* 15981 and MW2 strains, grown in MH supplemented with the corresponding antibiotic, were treated for 2 h at 37°C in agitation with 100 µg mL^−1^ proteinase K in 20 mM Tris (pH 7.5), 100 mM NaCl, 100 U mL^−1^ DNase I, and 10 mM sodium metaperiodate (NaIO_4_) in 50 mM sodium acetate buffer (pH 4.5), and ring formation and/or cell aggregation disruption was observed.

Purified aggregates were treated with the same concentrations of proteinase K and DNase I, incubated 2 h at 37°C, and directly observed under the microscope. The amyloidogenic nature of the aggregates was assessed by measuring emission of fluorescence upon ThT binding. Aggregates in PBS were incubated with 32 μΜ ThT for 30 min and visualized directly under the fluorescence microscope.

### Statistical analyses

Statistical significance was determined with the *t*-test by using GraphPad Prism software. Results were considered significant if the *P*-value was <0.05, which was indicated with asterisks in the corresponding plots.

## Data Availability

The raw sequencing reads generated in this study are available at the Sequence Read Archive (SRA) under the BioProject accession number PRJNA1231782 (https://www.ncbi.nlm.nih.gov/bioproject/PRJNA1231782). The raw mass spectrometry proteomics data have been deposited to the ProteomeXchange Consortium via the PRIDE partner repository with the data set identifier PXD061402 (https://www.ebi.ac.uk/pride/archive/projects/PXD061402). The genome wide transcriptomic, translatomic and peptidomic maps analyzed in this study were compiled in a web browser available at https://rnamaps.unavarra.es/RIBOseq/JBrowse-1.16.10/index.html. Other data or material that support the findings of this study can be made available by the corresponding author upon request.

## SUPPLEMENTAL MATERIAL

Supplemental material mbio.01856-25-s0001.pdf Supplemental methods, Tables S3 to S5, Figures S1 to S7, and references.

Table S1 mbio.01856-25-s0002.xlsx Identification of SaSPs smaller than 50 aa.

Table S2 mbio.01856-25-s0003.xlsx Mass spectrometry analysis of excised gel bands.

ASM does not own the copyrights to Supplemental Material that may be linked to, or accessed through, an article. The authors have granted ASM a non-exclusive, world-wide license to publish the Supplemental Material files. Please contact the corresponding author directly for reuse.
