# Supplementary material for: Multiomic data analyses unveiled a novel phenol-soluble modulin that induces trapping of extracellular lipases at the surface of Staphylococcus aureus cells
Source: mBio. 2025 Sep 29;16(11):e01856-25. doi: 10.1128/mbio.01856-25 (PMC12607899; doi:10.1128/mbio.01856-25)
Supplement: Supplemental material — Supplemental methods, Tables S3 to S5, Figures S1 to S7, and references. [file mbio.01856-25-s0001.pdf]

Supplementary Data for

Multiomic data analyses unveiled a novel phenol-soluble modulin that induces trapping of extracellular lipases at *Staphylococcus aureus* cells

Ane Muruzabal-Galarza[1], Arancha Catalan-Moreno[1], Pedro Dorado-Morales[1], Coral García-Gutiérrez[1], Miriam Torguet[1], Isabelle Caldelari[2], Carlos J. Caballero[1], Jaione Valle[1] and Alejandro Toledo-Arana[1]*

[1]Instituto de Agrobiotecnología, Consejo Superior de Investigaciones Científicas (IdAB-CSIC)-Gobierno de Navarra. 31192-Mutilva, Navarra, Spain.

[2]Université de Strasbourg, CNRS, Architecture et Réactivité de l'ARN, UPR9002, F-67000-Strasbourg, France

*Corresponding author: Alejandro Toledo-Arana
E-mail: a.toledo.arana@csic.es
Phone: +34 948 16 9752
Laboratory of Bacterial Gene Regulation
Instituto de Agrobiotecnología
CSIC-Gobierno de Navarra
Avda. de Pamplona 123
31192-Mutilva, Navarra
Spain

This PDF file includes:

Supplementary Methods

Supplementary Tables S3 to S5

Supplementary Figures S1 to 7

Supplementary References

SUPPLEMENTARY METHODS

Construction of plasmids used for small protein RBS validations

For analysing the expression of a subset of selected SaSPs, translational gene fusions were constructed in the pCN57^{+1} plasmid (1). To do that, the first three codons of each sORF alongside 31 nucleotides upstream of the translation initiation site, including the putative RBS, were fused in-frame with an ATG-less green fluorescent protein (GFP*)* gene by PCR amplification of part of the *gfp* gene with the corresponding forward oligonucleotide and the reverse oligonucleotide 238, using as template the pCN57^{+1} plasmid. For the generation of pCN57^{+1}_RBS-SaSP_006, _025, _028, _031, _032, _ _051, _085, _087 and _088 plasmids, the forward oligonucleotides 228, 237, 330, 331, 226, 248, 329, 245, 348 and 225 were used, respectively. The PCR products were digested using EcoRI and NcoI enzymes and ligated into the pCN57^{+1} plasmid digested with the same enzymes.

Construction of GFP translational reporter plasmids for analysing SAL1 expression regulation by LspU translation

For the generation of pHRG-P$_{sal1}$-LspU-GFP and pHRG-P$_{sal1}$-LspU-SAL1-GFP plasmids, the *sal1* promoter region, 5´UTR and complete *lspU* regions or *sal1* three first codons, respectively, were amplified using oligonucleotides 307 in both cases and 310 or 311 for each, respectively. For obtaining pHRG-P$_{sal1}$-LspU^{STOP}-GFP and pHRG-P$_{sal1}$-LspU^{STOP}-SAL1-GFP plasmids, the base substitution to generate a stop codon in the fifth codon was introduced by overlapping PCRs using oligonucleotides 307 and 308 and other with 309 and 310 or 311, respectively. The PCR fragments were cloned into pHRG using SphI and SpeI restriction sites.

Construction of plasmids expressing wild type and mutant *lspU* versions

For generating pHRG-LspU and pHRG-LspU^{STOP}plasmids, wild type *lspU* region was amplified by PCR using 349 and 356 oligonucleotides and *S. aureus* DNA, while mutant *lspU*, which carried a base substitution to generate a stop codon in the fifth codon, was generated by overlapping PCRs using oligonucleotide pairs 349 and 308 and 309 and 356, respectively. Both PCR fragments were digested using MunI and AscI restriction enzymes and ligated into pHRG plasmid digested with EcoRI and AscI.

Construction of tagged LspU expression plasmids

Aiming at facilitating the labelling of small proteins with the 3xFLAG tag, we generated the pHRF plasmid. Briefly, the 3xFLAG sequence was introduced by PCR amplification

using the oligonucleotides 1111 and 1112 and pHRG plasmid as template. The PCR fragment was digested with SpeI and NarI restriction enzymes and ligated into the pHRG plasmid digested with the same enzymes. As a result, the *gfp* sequence was replaced by the 3xFLAG sequence generating the pHRF plasmid.

The pHRF-LspU^{3xFLAG} plasmid was generated by PCR amplification of *lspU* region using the oligonucleotides 349 and 310, and *S. aureus* DNA as template. The PCR product was digested using MunI and SpeI, and ligated into pHRF plasmid digested with EcoRI and SpeI.

The pHRF-LspU^{FLAG} plasmid was constructed by substituting the 3xFLAG by the FLAG sequence using oligonucleotides 454 and 455, which both hybridize to simulate a DNA fragment carrying digested ends by SpeI and AscI restriction enzymes. Both oligonucleotides were annealed, and ligated into the pHRF-LspU^{3xFLAG} plasmid digested with SpeI and AscI.

The pHRG-LspU-GFP plasmid was constructed by amplifying the *lspU* region with oligonucleotide pairs 349 and 310, and *S. aureus* DNA as template. PCR product was digested with MunI and SpeI restriction enzymes and ligated into pHRG plasmid digested with EcoRI and SpeI.

Construction of LspU expression plasmids with tetracyline resistance gene

The pHRT plasmid is a pHRG-derived plasmid where erythromycin resistance (Erm^R) cassette was substituted by the tetracycline resistance (Tet^R) cassette. Briefly, the Tet^R cassette was digested from pCN36 plasmid (2) using XhoI and ApaI restriction enzyme and ligated into pHRG plasmid digested with the same enzymes. The pHRT-LspU^{FLAG} plasmid was constructed by amplifying *lspU* region using oligonucleotide pairs 349 and 603. The PCR fragment was digested with MunI and AscI and ligated into pHRT plasmid digested with EcoRI and AscI.

Constructions of pMAD plasmids used for chromosomal mutagenesis

The pMAD plasmids shown in Supplementary Table S2 were used to generate modifications in the chromosome of indicated strains, either to insert the 6xHis tag into *sal1* and *sal2* genes, change *sal2* gene to *sal1*^{6xHis} gene, fuse *mRFPmars* fluorescent reporter to *sal2* gene or mutate *sal1*, *sal2* and/or *psm*-encoding genes. Briefly, most of the mutant alleles were generated by overlapping PCRs using the oligonucleotides listed in Supplementary Table S3 and *S. aureus* DNA as template, otherwise it is explained below. PCR fragments were digested with the indicated enzymes and ligated into pMAD plasmid.

The pMAD-*sal2*-MARS plasmid was constructed by three PCR fragment ligation into pMAD. The first PCR fragment was generated by PCR amplification of a *sal2* region using the oligonucleotides 463 and 1113. The second PCR fragment includes the *mRFPmars* region (in frame with the previous gene), which was amplified using oligonucleotides 574 and 575 and USA300_TCH1516 *amy::*P_{SA1403}*-floA-mars* strain as template (3). The third PCR fragment including the downstream *sal2* region was amplified using the oligonucleotides 601 and 602 and *S. aureus* DNA as a template. The three PCR fragments were digested with BglII/SpeI, SpeI/AscI and AscI/EcoRI restriction enzyme pairs, respectively, and ligated into pMAD plasmid digested with BglII/EcoRI.
pMAD-exo-MARS was generated by generating a PCR fragment using oligonucleotides 810 and 811 from *S. aureus* as template, that corresponds to pre-pro sal2 region, leaving aureolysin cleavage site. This PCR fragment was digested with BglII and SpeI restriction enzyme and ligated into the pMAD_*sal2*-MARS digested with the same enzymes.

SUPPLEMENTARY TABLES

Table S3. Strains used in this study

Strains	Relevant characteristic(s)	BGR ID [a]	Source or reference
Escherichia coli			
XL1-Blue	Strain used for cloning experiments	1	Stratagene
IM01B	Strain used for cloning experiments	1837	(4)
Staphylococcus aureus			
15981	MSSA clinical isolate; biofilm positive; PNAG-dependent biofilm matrix	8	(5)
MW2	Community-acquired MRSA strain, isolated in 1998 in North Dakota, USA	10	(6)
USA300_TCH1516 *amy::P$_{SA1403}$-floA-mars*	USA300_TCH1516 strain expressing the FloA-MARS protein fusion from the chromosome		(3)
15981 pCN57^{+1}	15981 strain carrying the pCN57^{+1} plasmid	322	(1)
15981 pTL-81 WT	15981 strain carrying the pTL WT plasmid	938	(1)
15981 pTL-81_STOP34	15981 strain carrying the pTL STOP^{34} plasmid	947	(1)
15981 pRBS-SaSP_006	15981 strain carrying pRBS-SaSP_006 plasmid	2515	This study
15981 pRBS-SaSP_025	15981 strain carrying pRBS-SaSP_025 plasmid	2502	This study
15981 pRBS-SaSP_028	15981 strain carrying pRBS-SaSP_028 plasmid	2535	This study
15981 RBS-SaSP_031	15981 strain carrying pRBS-SaSP_031 plasmid	2518	This study
15981 pRBS-SaSP_032	15981 strain carrying pRBS-SaSP_032 plasmid	2510	This study
15981 pRBS-SaSP_043	15981 strain carrying pRBS-SaSP_043 plasmid	2449	This study
15981 pRBS-SaSP_051	15981 strain carrying pRBS-SaSP_051 plasmid	2519	This study
15981 pRBS-SaSP_085	15981 strain carrying pRBS-SaSP_085 plasmid	2503	This study
15981 pRBS-SaSP_087	15981 strain carrying pRBS-SaSP_087 plasmid	2605	This study
15981 pRBS-SaSP_088	15981 strain carrying pRBS-SaSP_088 plasmid	2516	This study
15981 pHRG-P$_{sal1}$-LspU-GFP	15981 strain carrying the pHRG-P$_{sal1}$-LspU-GFP plasmid	2438	This study
15981 pHRG-P$_{sal1}$-LspU-SAL1-GFP	15981 strain carrying thepHRG-P$_{sal1}$-LspU-SAL1-GFP plasmid	2440	This study
15981 pHRG-P$_{sal1}$-LspU^{STOP}-GFP	15981 strain carrying the pHRG-P*sal1*-LspU^{STOP}-GFP plasmid	2439	This study
15981 pHRG-P$_{sal1}$-LspU^{STOP}-SAL1-GFP	15981 strain carrying the pHRG-P*sal1*-LspU^{STO}-SAL1-GFP plasmid	2441	This study
15981 pHRG-LspU	15981 strain carrying the pHRG-LspU plasmid	2623	This study
15981 pHRG-LspU^{STOP}	15981 strain carrying the pHRG-LspU^{STOP} plasmid	2625	This study
15981 pES	15981 strain carrying pES plasmid	370	This study
MW2 pHRG-LspU	MW2 strain carrying the pHRG-LspU plasmid	2624	This study
MW2 pHRG-LspU^{STOP}	MW2 strain carrying the pHRG-LspU^{STOP} plasmid	2626	This study
MW2 pES	MW2 strain carrying pES plasmid	620	This study
MW2 pHRF-LspU^{3xFLAG}	MW2 strain carrying thepHRF-LspU^{3xFLAG} plasmid	2801	This study
MW2 pHRG-LspU^{FLAG}	MW2 strain carrying the MW2 pHRG pHRG-LspU^{FLAG} plasmid	2783	This study
MW2 pHRG-LspU-gfp	MW2 strain carrying the pHRG-LspU^{gfp} plasmid	2884	This study
MW2 Δ*sal1*	MW2 strain with the deletion of the*sal1* operon	2653	This study
MW2 Δ*sal1* pHRG-LspU	MW2 Δ*sal1* strain carrying the pHRG-LspU plasmid	2733	This study
MW2 Δ*sal1* pHRG-LspU^{STOP}	MW2 Δ*sal1* strain carrying the pHRG-LspU^{STOP} plasmid	2734	This study

Continued in the following page

Table S3. *Continued*

**Strains**	**Relevant characteristic(s)**	**BGR ID** [a]	**Source or reference**
MW2 Δ*sal2*	MW2 strain with the deletion of the *sal2* gene	2655	This study
MW2 Δ*sal2* pHRG-LspU	MW2 Δ*sal2* strain carrying the pHRG-LspU plasmid	2731	This study
MW2 Δ*sal2* pHRG-LspU^{STOP}	MW2 Δ*sal2* strain carrying the pHRG-LspU^{STOP} plasmid	2732	This study
MW2 Δ*sal1* Δ*sal2*	MW2 strain with the deletion of both *sal1* operon and *sal2* gene	2686	This study
MW2 Δ*sal1* Δ*sal2* pHRG-LspU	MW2 Δ*sal1* Δ*sal2* strain carrying the pHRG-LspU plasmid	2735	This study
MW2 Δ*sal1* Δ*sal2* pHRG-LspU^{STOP}	MW2 Δ*sal1* Δ*sal2* strain carrying the pHRG-LspU^{STOP} plasmid	2736	This study
MW2 *sal1*^{6xHis}	MW2 strain expressing the 6xHis-tagged SAL1 protein from the chromosome	2832	This study
MW2 *sal1*^{6xHis} pHRG-LspU	MW2 *sal1*^{6xHis} strain carrying the pHRG-LspU plasmid	2839	This study
MW2 *sal1*^{6xHis} pHRG-LspU^{STOP}	MW2 *sal1*^{6xHis} strain carrying the pHRG-LspU^{STOP} plasmid	2840	This study
MW2 *sal2*^{6xHis}	MW2 strain expressing the 6xHis-tagged SAL2 protein from the chromosome	2831	This study
MW2 *sal2*^{6xHis} pHRG-LspU	MW2 *sal2*^{6xHis} strain carrying the pHRG-LspU plasmid	2837	This study
MW2 *sal2*^{6xHis} pHRG-LspU^{STOP}	MW2 *sal2*^{6xHis} strain carrying the pHRG-LspU^{STOP} plasmid	2838	This study
MW2 *sal2*^{6xHis} Δ*sal1*	MW2 *sal2*^{6xHis} strain with the deletion of the *sal1* operon	2885	This study
MW2 *sal2*^{6xHis} Δ*sal1* pHRG-LspU	MW2 *sal2*^{6xHis} Δ*sal1* strain carrying the pHRG-LspU plasmid	2886	This study
MW2 *sal2*^{6xHis} Δ*sal1* pHRG-LspU^{STOP}	MW2 *sal2*^{6xHis} Δ*sal1* strain carrying the pHRG-LspU^{STOP} plasmid	2887	This study
MW2 Δ*sal1* P_{sal2}::*sal1*^{6xHis}	MW2 Δ*sal1* strain expressing the 6xHis-tagged SAL1 protein under the control of the P_{sal2} promoter from the chromosome.	2878	This study
MW2 Δ*sal1* P_{sal2}::*sal1*^{6xHis} pHRG-LspU	MW2 Δ*sal1* P_{sal2}::*sal1*^{6xHis} strain carrying the pHRG-LspU plasmid	2879	This study
MW2 Δ*sal1* P_{sal2}::*sal1*^{6xHis} pHRG-LspU^{STOP}	MW2 Δ*sal1* P_{sal2}::*sal1*^{6xHis} strain carrying the pHRG-LspU^{STOP} plasmid	2880	This study
USA300 LAC Δ*psmα*β*δ*	USA300 LAC strain with deletions in the *psm-α* and *psm-β* operons, and a base subtitution in *hld* gene to change its start codon ATG to ATT avoiding the translation of *psmδ*	3059	(7)
USA300 LAC Δ*psmα*β*δ* Δ*pmtABCD* Δ*abcA*	USA300 LAC Δ*psmα*β*δ* strain with deletions in the *pmtABCD* operon and *abcA* gene	3060	(7)
USA300 LAC Δ*psmα*β*δ* pHRT-LspU^{FLAG}	USA300 LAC Δ*psmα*β*δ* strain carrying the pHRT-LspU^{FLAG} plasmid.	3156	This study
USA300 LAC Δ*psmαβδ* Δ*pmtABCD* Δ*abcA* pHRT-LspU^{FLAG}	USA300 LAC Δ*psmα*β*δ* Δ*pmtABCD* Δ*abcA* strain carrying the pHRT-LspU^{FLAG} plasmid	3158	This study
MW2 *sal2-MARS*	MW2 strain expressing *sal2* gene fused to *mRFPmars* red fluorescent from the chromosome	2938	This study
MW2 *sal2-MARS* pHRG-LspU	MW2 *sal2-MARS* strain carrying the pHRG-LspU plasmid	2943	This study
MW2 *sal2-MARS* pHRG-LspU^{STOP}	MW2 *sal2-MARS* strain carying the pHRG-LspU^{STOP} plasmid	2945	This study
MW2 *exo-MARS*	MW2 strain expressing the *exo-MARS* chimeric gene from the chromosome. The gene region encoding the SAL2 mature lipase was replaced by the mRFPmars	3176	This study
MW2 exo-*MARS* pHRG-LspU	MW2 *exo-MARS* strain carrying the pHRG-LspU plasmid	3177	This study
MW2 exo-*MARS* pHRG-LspU^{STOP}	MW2 *exo-MARS* strain carrying the pHRG-LspU^{STOP} plasmid	3178	This study

Continued in the following page

Table S3. *Continued*

Strains	Relevant characteristic(s)	BGR ID [a]	Source or reference
MW2 Δ*psmαβδ*	MW2 strain with deletions in the *psm-α* and *psm-β* operons, and a base subtitution in the *psmδ* gene to change its ATG start codon from to ATT avoiding the translation	3311	This study
MW2 Δ*psmαβδ* pHRG-LspU	MW2 Δ*psmαβδ* strain carrying the pHRG-LspU plasmid	3337	This study
MW2 Δ*psmα*β*δ* pHRG-LspU^{STOP}	MW2 Δ*psmαβδ* strain carrying the pHRG-LspU^{STOP} plasmid	3338	This study
MW2 Δ*psmα*β*δ sal2-MARS*	MW2 Δ*psmαβδ* strain expressing from the chromosome the *sal2* gene fused to *mRFPmars* red fluorescent gene	3364	This study
MW2 Δ*psmαβδ sal2-MARS* pHRG-LspU	MW2 Δ*psmα*β*δ sal2-MARS* strain carrying the pHRG-LspU plasmid	3379	This study
MW2 Δ*psmαβδ sal2-MARS* pHRG-LspU^{STOP}	MW2 Δ*psmα*β*δ sal2-MARS* strain carrying the pHRG-LspU^{STOP} plasmid	3380	This study

[a] Identification number of the strains stored at the Laboratory of Bacterial Gene Regulation.

Table S4. Plasmids used in this study

Plasmids	Relevant characteristic(s)	Source or reference
pCN57^{+1}	pCN57 plasmid carrying the modified *PblaZ* promoter region to express mRNAs from their native transcriptional start site. Amp^{R} in *E. coli*. Erm^{R} in *S. aureus*.	(1)
pTL-81_WT	pHRG plasmid in which P*hyper* was replaced by *Pblaz*$^{+1}$ and the *sbrB* coding sequence was fused to the ATG-less *gfp* reporter gene. Amp^{R} in *E. coli*. Erm^{R} in *S. aureus*.	(1)
pTL-81_STOP^{34}	pTL WT plasmid where the adenine at position 34 of the *sbrB* was substituted by a thymine generating a stop codon. Amp^{R} in *E. coli*. Erm^{R} in *S. aureus*.	(1)
pRBS-SaSP_006	pCN57^{+1} translation-reporter plasmid carrying the RBS region of SaSP_006 fused to the ATG-less *gfp* reporter gene.	This study
pRBS-SaSP_025	pCN57^{+1} translation-reporter plasmid carrying the RBS region of SaSP_025 fused to the ATG-less *gfp* reporter gene.	This study
pRBS-SaSP_028	pCN57^{+1} translation-reporter plasmid carrying the RBS region of of SaSP_028 fused to the ATG-less *gfp* reporter gene.	This study
pRBS-SaSP_031	pCN57^{+1} translation-reporter plasmid carrying the RBS region of SaSP_031 fused to the ATG-less *gfp* reporter gene.	This study
pRBS-SaSP_032	pCN57^{+1} translation-reporter plasmid carrying the RBS region of SaSP_032 fused to the ATG-less *gfp* reporter gene.	This study
pRBS-SaSP_043	pCN57^{+1} translation-reporter plasmid carrying the RBS region of SaSP_043 fused to the ATG-less *gfp* reporter gene.	This study
pRBS-SaSP_051	pCN57^{+1} translation-reporter plasmid carrying the RBS region of SaSP_051 fused to the ATG-less *gfp* reporter gene.	This study
pRBS-SaSP_085	pCN57^{+1} translation-reporter plasmid carrying the RBS region of SaSP_085 fused to the ATG-less *gfp* reporter gene.	This study
pRBS-SaSP_087	pCN57^{+1} translation-reporter plasmid carrying the RBS region of SaSP_087 fused to the ATG-less *gfp* reporter gene.	This study
pRBS-SaSP_088	pCN57^{+1} translation-reporter plasmid carrying the RBS region of SaSP_088 fused to the ATG-less *gfp* reporter gene.	This study
pHRG	pCN47 plasmid containing the P*hyper* constitutive promoter, *icaR* RBS and *gfp* reporter gene for fluorescent translational reporter assays. Amp^{R} in *E. coli*. Erm^{R} in *S. aureus.*	(8)
pHRF	pHRG plasmid where the *gfp* reporter gene has been substituted by the 3xFLAG sequence. Amp^{R} in *E. coli*. Erm^{R} in *S. aureus*.	This study
pHRG-P$_{sal1}$-LspU-GFP	pHRG translation-reporter plasmid carrying the wild type *lspU* gene fused to the *gfp* gene under the control of its native P$_{sal1}$ promoter.	This study
pHRG-P$_{sal1}$-LspU-SAL1-GFP	pHRG translation-reporter plasmid carrying the wild type *lspU* sORF and the translational *sal1-gfp* gene fusion under the control of its native P$_{sal1}$ promoter.	This study
pHRG-P$_{sal1}$-LspU^{STOP}-GFP	pHRG translation-reporter plasmid carrying a mutated *lspU* sORF form (the fifth codon is substituted to a stop codon) fused to the *gfp* gene under the control of its native P$_{sal1}$ promoter.	This study
pHRG-P$_{sal1}$-LspU^{STOP}-SAL1-GFP	pHRG translation-reporter plasmid carrying the mutated *lspU* sORF and the translational *sal1-gfp* fusion reporter under the control of its native P$_{sal1}$ promoter.	This study
pES	pCN47 plasmid containing the promoter of blaZ gene (PblaZ) from pCN57 plasmid	(9)
pHRG-LspU	pHRG plasmid expressing the wild type *lspU* sORF under the control of the P$_{hyper}$ promoter	This study
pHRG_LspU^{STOP}	pHRG plasmid expressing the mutated *lspU* sORF version under the control of the P$_{hyper}$ promoter. The fifth codon is substituted to a stop codon.	This study
pHRF-LspU^{3xFLAG}	pHRG plasmid expressing the *lspU*^{3xFLAG} tagged gene under the control of the P$_{hyper}$ promoter. The 3xFLAG epitope was fused to *lspU* C-terminal end.	This study
pHRF-LspU^{FLAG}	pHRG plasmid expressing the *lspU*^{FLAG} tagged gene under the control of the P$_{hyper}$ promoter. The FLAG epitope was fused to *lspU* C-terminal end.	This study

Continued in the following page

Table S4. *Continued*

Plasmids	Relevant characteristic(s)	Source or reference
pHRG-LspU-GFP	pHRG translation-reporter plasmid carrying the wild type *lspU* gene fused to the ATG-less *gfp* gene under the control of the P_{hyper} promoter.	This study
pCN36	*E. coli* and *S. aureus* shuttle vector, which contains tetracycline resistance gene *tetA*(M) as selective marker for *S. aureus* strains. Amp^R in *E. coli*. Tet^R in *S. aureus*	(2)
pHRT	pHRG-like plasmid with the erythromycin resistance gene substituted to a tetracycline resistance gene from pCN36 plasmid. Amp^R in *E. coli*. Tet^R in *S. aureus*	This study
pHRT_LspU^{FLAG}	pHRT plasmid expressing the *lspU*^{FLAG} tagged gene under the control of the P_{hyper} promoter	This study
pMAD	*E. coli*-*S. aureus* shuttle vector with a thermosensitive origin of replication for Gram-positive bacteria. It contains the *bgaB* gene that encodes β-galactosidase under the control of a constitutive promoter. Amp^R in *E. coli*. Erm^R in *S. aureus*	(10)
pMAD-Δ*sal1*	pMAD containing the allele for the deletion of the *sal1* gene	This study
pMAD-Δ*sal2*	pMAD containing the allele for the deletion of the *sal2* gene	This study
pMAD-*sal1*^{6xHis}	pMAD containing the allele for labelling *sal1* gene with the 6xHis tag	This study
pMAD-*sal2*^{6xHis}	pMAD containing the allele for labelling *sal2* gene with the 6xHis tag	This study
pMAD-P_{sal2}::*sal1*^{6xHis}	pMAD containing the allele for the substitution of *sal2* gene to *sal1*^{6xHis} gene	This study
pMAD-*sal2*-MARS	pMAD containing the allele for the fusion of *sal2* gene to the MARS red fluorescent protein	This study
pMAD-exo-MARS	pMAD containing the allele for fusion *sal2* pre-pro regions (without the region coding for the mature protein) to the MARS red fluorescent protein	This study
pMAD-Δ*psmα*	pMAD containing the allele for the deletion of the PSM alpha operon	This study
pMAD-Δ*psmβ*	pMAD containing the allele for the deletion of the PSM beta operon	This study
pMAD-Δ*psmδ*	pMAD containing the allele for the substitution of the start codon of the *psmδ* gene, ATG, to ATT, impeding its translation initiation	This study

Table S5. Oligonucleotides used in this study

BGR ID	Sequence [a]	Restriction enzyme
Construction of plasmids used for RBSs validation		
228	GG*GAATTC*GTCAAATTAAACAATAGAAAGGATGGTCATTATGAGTGCTA<br>CTAGTAAAGGAGAAGAACTTTTCA	EcoRI
237	GG*GAATTC*TATATAAATAATTGTTTTAGGGAGAATAATCGTGACTGCAAC<br>TAGTAAAGGAGAAGAACTTTTCA	EcoRI
330	GG*GAATTC*TATATAAATAATTGTTTTAGGGAGAATAATCGTGACTGCAAC<br>TAGTAAAGGAGAAGAACTTTTCA	EcoRI
331	GG*GAATTC*TACGAGAGACTGAAAAAGGTGGGGGAGTTGAATGATAAAT<br>ACTAGTAAAGGAGAAGAACTTTTCA	EcoRI
226	GG*GAATTC*ACAATTAAAATTAAGTTAAAGGAGGGACGTTATGTTACATA<br>CTAGTAAAGGAGAAGAACTTTTCA	EcoRI
248	GG*GAATTC*TACAAAGGTTATTCATTTGGAGGCACACAAAATGCAAAATA<br>CTAGTAAAGGAGAAGAACTTTTCA	EcoRI
329	GG*GAATTC*TGTTAAAATATTTTTAACGAGGTGAATTAAATTGAAATTTAC<br>TAGTAAAGGAGAAGAACTTTTCA	EcoRI
245	GG*GAATTC*TTTCAACACATCTTAGAAAGGAGTTTGAATGATGAAAAAAA<br>CTAGTAAAGGAAAAGAACTTTTCA	EcoRI
348	GG*GAATTC*ATAATTTATAAAGTAAAGGGAGGAATTAATAATGACTGCAA<br>CTAGTAAAGGAGAAGAACTTTTCA	EcoRI
225	GG*GAATTC*GTCAAATTAAACAATAGAAAGGATGGTCATTATGAGTGCTA<br>CTAGTAAAGGAGAAGAACTTTTCA	EcoRI
238	AAGTGTTGG*CCATGG*AACAG	NcoI
Construction of GFP translational reporter plasmids for analysing SAL1 expression		
307	GG*GCATGC*AAGCCCCAAGCATGTAAATA	SphI
310	GG*ACTAGT*GATAAACAAATTGATAATTATTCGAGG	SpeI
311	GG*ACTAGT*ACTTTTCATCATTGTCAGCAC	SpeI
308	TACCAAGCACTCATAATGACC	
309	GGTCATTATGAGTGCTTGGTAAAGTAAATTATTTGAGTTTATTCCTCG	
Construction of plasmids expressing wild type and mutant *lspU* versions		
349	GG*CAATTG*AATAATTTGAAATGATTAGCGTATAC	MunI
356	GG*GGCGCGCC*TTAGATAAACAAATTGATAATTATTCGAGG	AscI
308	TACCAAGCACTCATAATGACC	
309	GGTCATTATGAGTGCTTGGTAAAGTAAATTATTTGAGTTTATTCCTCG	
Construction of tagged *lspU* expression plasmids		
1111	*ACTAGT*GACTACAAAGACCATGACGGTGATTATAAAGATCATGATATCG<br>ACTACAAAGATGACGACGATAAATAAGGCGCGCCTATTCTAAATG	SpeI
1112	AAA*GGCGCC*TGTCACTTTGC	NarI
307	GG*GCATGC*AAGCCCCAAGCATGTAAATA	SphI
310	GG*ACTAGT*GATAAACAAATTGATAATTATTCGAGG	SpeI
454	CTAGTGACTACAAAGATGACGACGATAAATAAGG	
455	CGCGCCTTATTTATCGTCGTCATCTTTGTAGTCA	
349	GG*CAATTG*AATAATTTGAAATGATTAGCGTATAC	MunI
Construction of *lspU* expression plasmids with the tetracyline resistance gene		
349	GG*CAATTG*AATAATTTGAAATGATTAGCGTATAC	MunI
603	CAGTATTTATTATGCATTTAGAATA	
Constructions of pMAD plasmids used for chromosomal mutagenesis		
pMAD-Δ*sal1*		
Primers for construction of mutant allele		
372	GG*CCCGGG*ATGCATCCCGAAAGTTAGAC	SmaI
373	GCCTCGCCAATGTTTTACTTTTTAATTTGACGAGAAAGCG	
374	AAGTAAAACATTGGCGAGGC	
375	GG*AGATCT*CATTCTAGACTCATTGTTTGC	BglII
Primers for checking Δ*sal1* mutation in the *S. aureus* chromosome		
376	ACGATTCAAATGATTAGTGC	
377	CTGAAATAGAAATGTCATAGCC	

Continued in the following page

Table S5. *Continued*

BGR ID	Sequence [a]	Restriction enzyme
pMAD-Δ*sal2*		
Primers for construction of mutant allele		
378	GG*AGATCT*GTTGTTACCATTAATCGTAAAAGG	BglII
379	GTATTTTTTTGACCCCCTCT	
380	AGAGGGGGTCAAAAAAATACATTCATCTTCTGAATTTAATATGC	
381	GG*CCCGGG*GCACAGGTAAATGGATAAATATG	SmaI
Primers for checking Δ*sal2* mutation in the *S. aureus* chromosome		
382	AACATGATCGACGAAAATAG	
383	GTTTTTTAATCTACGTTTATGCAC	
pMAD-*sal1*^{6xHis}		
Primers for construction of mutant allele		
439	GG*CCCGGG*GTCTAAAACAGAAGCCAAATG	SmaI
440	TTAGTGGTGATGGTGATGATGTGCTTGCTTAGTATCAGTCA	
441	CACATCATCACCATCACCACTAATTTATAAAGTAAAGGGAGGAATTAAT	
1088	GG*AGATCT*GGCATGATATTGCGTGAATTC	BglII
Primers for checking *sal1*^{6xHis} modification in the *S. aureus* chromosome		
376	ACGATTCAAATGATTAGTGC	
442	GCACATCATCACCATCACCAC	
377	CTGAAATAGAAATGTCATAGCC	
pMAD-*sal2*^{6xHis}		
Primers for construction of mutant allele		
463	*AGATCT*ACGACTTATACAGGTGTATCATCTCATA	BglII
1089	ACTTGCTTTCAATTGTGTTCC	
381	GG*CCCGGG*GCACAGGTAAATGGATAAATATG	SmaI
446	GGAACACAATTGAAAGCAAGTCATCATCACCATCACCACTAAATTCATCTTCTGAATTTAATATGC	
Primers for checking *sal2*^{6xHis} modification in the *S. aureus* chromosome		
383	GTTTTTTAATCTACGTTTATGCAC	
442	GCACATCATCACCATCACCAC	
pMAD-P$_{sal2}$::*sal1*^{6xHis}		
Primers for construction of mutant allele		
464	*GGCCATG*GGTTGTTACCATTAATCGTAAAAGG	NcoI
1090	ATAATCACCTCAAAAGTATTTTTTTG	
443	AGAGGGGGTCAAAAAAATACTTTTGAGGTGATTATATGAAAAGTCAAAATAAGTATAGTATTC	
440	TTAGTGGTGATGGTGATGATGTGCTTGCTTAGTATCAGTCA	
444	ATCATCACCATCACCACTAAATTCATCTTCTGAATTTAATATGC	
465	GG*GTCGAC*GCACAGGTAAATGGATAAATATG	SalI
Primers for checking P$_{sal2}$::*sal1*^{6xHis} modification in the *S. aureus* chromosome		
382	AACATGATCGACGAAAATAG	
383	GTTTTTTAATCTACGTTTATGCAC	
pMAD-*sal2*-MARS		
Primers for construction of mutant allele		
463	*AGATCT*ACGACTTATACAGGTGTATCATCTCATA	BglII
1113	AAAA*ACTAGT*ACTTGCTTTCAATTGTGTTCC	SpeI
574	AAAA*ACTAGT*GCATCATCAGAAGATG	SpeI
575	AAAA*GGCGCGCC*TTATCCTGCACCTGTTGAA	AscI
601	GG*CGCG*CCATTCATCTTCTGAATTTAATATGC	AscI
602	*GAATTC*TAATGGCGATTATGATGTTCCC	EcoRI
Primers for checking *sal2*-MARS modification in the *S. aureus* chromosome		
382	AACATGATCGACGAAAATAG	
383	GTTTTTTAATCTACGTTTATGCAC	

Continued in the following page

Table S5. *Continued*

BGR ID	Sequence [a]	Restriction enzyme
pMAD-exo-MARS		
Primers for construction of mutant allele		
810	AAA*AGATCT*TGAACGTCAAAATGATCAG	BglII
811	AAA*ACTAGT*TTTGTTAAGTGGTTGTACTTG	SpeI
Primers for checking pMAD-exo-MARS modification in the *S. aureus* chromosome		
382	AACATGATCGACGAAAATAG	
383	GTTTTTTAATCTACGTTTATGCAC	
pMAD-Δ*psmα*		
Primers for construction of mutant allele		
640	*GGATCC*ATAGTTTTGATAAAGCAGAAATT	BamHI
641	AGCGAATTGAATACTTAAAATTCTCAG	
643	*CCATGG*TCTAATCTCTCGCATAATTGC	NcoI
642	TTTTAAGTATTCAATTCGCTGATGCCAGCGATGATACCCATTAAGA	
Primers for checking pMAD-Δ*psmα* modification in the *S. aureus* chromosome		
639	AGGTGTTCTTAGGATCTTTTTGTT	
644	AAGCGCAATAACAAACAGTGA	
pMAD-Δ*psmβ*		
Primers for construction of mutant allele		
646	*CCATGG*TCATACTATATTACTGAAATTCAAAACAAAA	NcoI
647	TGAAAACACTCCTTAAAATTTAAATTTG	
649	*GGATCC*TTAGTAACAATATATAAATCATGTCTTTTCG	BamHI
648	ATTTAAATTTTAAGGAGTGTTTTCATATAATAACTAATATTCTTTAAAATA AACTG	
Primers for checking pMAD-Δ*psmβ* modification in the *S. aureus* chromosome		
645	TTACTGTATTTTCGGGGCTTAT	
650	TGATTTAACCTTCTCTTTTGCGT	
pMAD-Δ*psmδ*		
Primers for construction of mutant allele		
652	*GGATCC*GACTTATTCATATATTTTAACGGC	BamHI
653	ATTGCACAAGATATCATTTCAACA	
655	*CCATGG*AAGAAGGTGCATGTGCA	NcoI
654	TTGTTGAAATGATATCTTGTG*CAATTG*AAATCACTCCT	
Primers for checking pMAD-Δ*psmδ* modification in the *S. aureus* chromosome		
651	CATTTCGCCCTTTGCAAATGAATG	
656	TAAAAGTATAAATAGTATAATACTTTCTACATAAC	

[a] Restriction enzymes and tag sequences are indicated in italic and underlined, respectively

SUPPLEMENTARY FIGURES

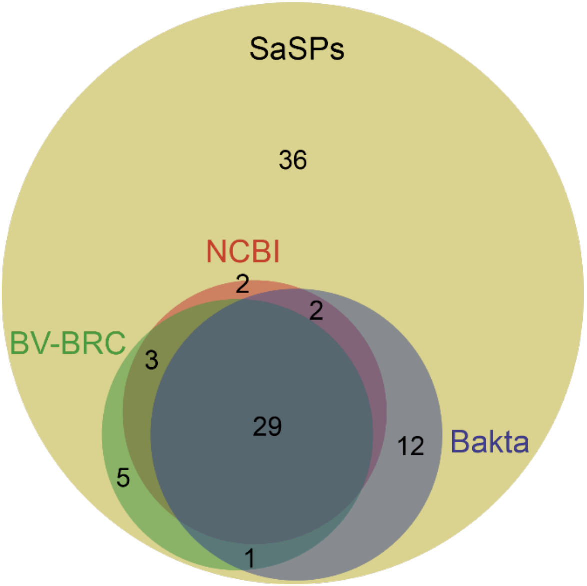

Figure S1. Identification of novel *S. aureus* small proteins (SaSPs). Venn diagram showing the number of unannotated and annotated SaSPs by the NCBI staff (https://www.ncbi.nlm.nih.gov/nuccore/NC_007795.1/), the Bacterial and Viral Bioinformatics Resource Center (BV_BRC, https://www.bv-brc.org/view/Genome/93061.5) (11, 12), and the Bakta web application (https://bakta.computational.bio) (13).

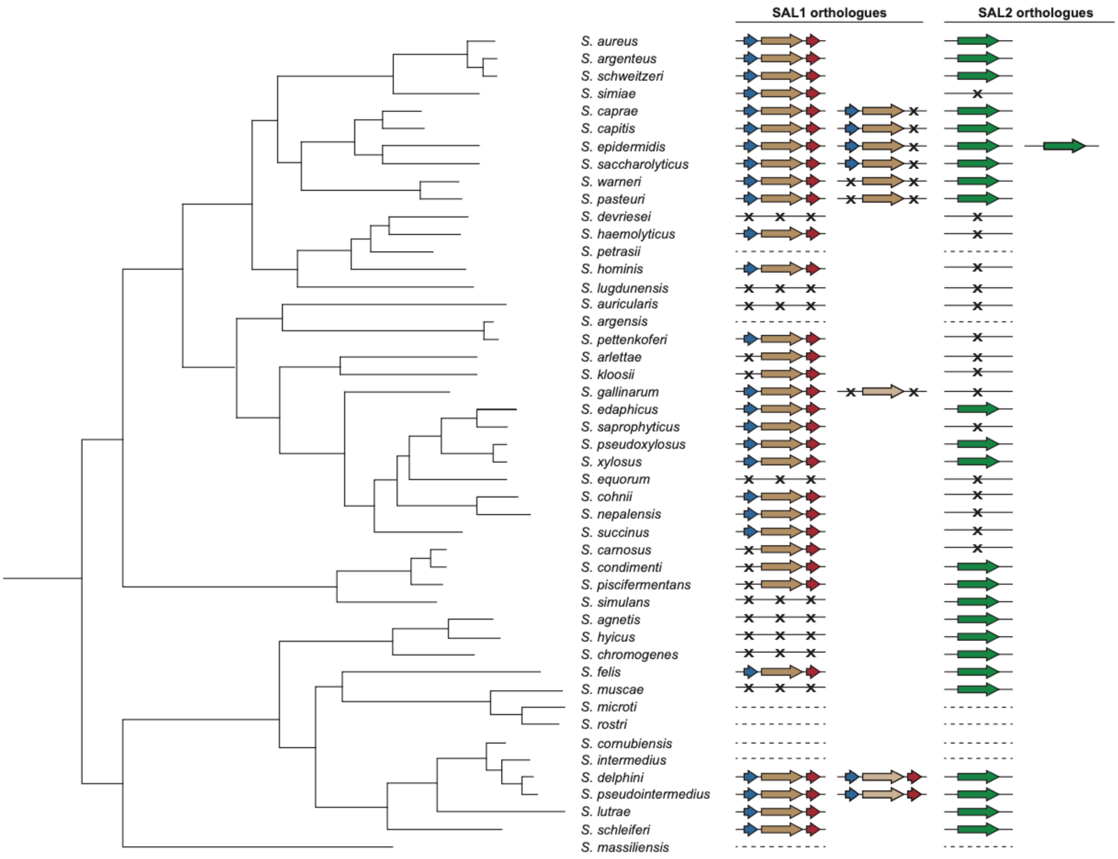

Figure S2. Distribution of *lspU* and *lspD* sORFs among the members of the genus *Staphylococcus*. The left panel shows the phylogenetic tree including the most representative members of the genus *Staphylococcus* adapted from Madhaiyan *et al* (14). The right panel shows the gene content in the genome of the corresponding representative species. Blue and red arrows represent the LspU and LspD sORFs. Brown and green arrows represent the SAL1 and SAL2 lipase orthologues, respectively. X, represents missing genes. Gene copies are shown in a second column. Dotted lines indicate that gene content was not analysed due to unavailable complete genome sequences.

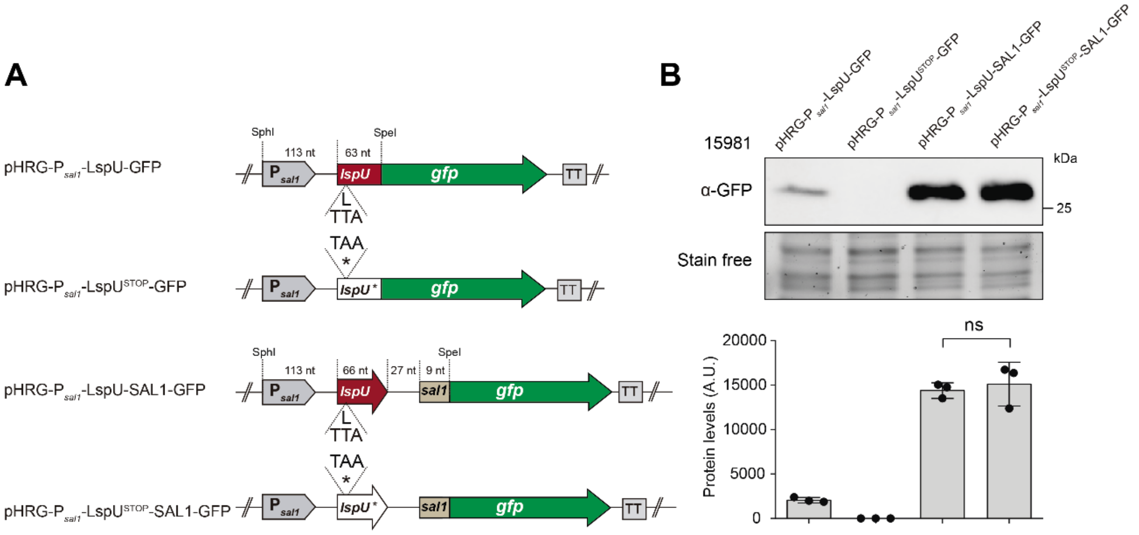

Figure S3. Translation of LspU does not affect the production of SAL1. (**A**) Schematic representation of the translational fluorescent reporter plasmids to evaluate gene expression. The base substitution in the fifth codon of LspU to generate a stop codon is indicated. The expression of chimeric genes is under the control of the native *sal1* promoter (P_{sal1}). Western blots showing the GFP protein levels in the protein extracts of strain 15981 carrying plasmids pHRG-P_{sal1}-LspU-GFP, pHRG-P_{sal1}-LspU^{STOP}-GFP, pHRG-P_{sal1}-LspU-SAL1-GFP and pHRG-P_{sal1}-LspU^{STOP}-SAL1-GFP. Membranes were developed using a combination of anti-GFP antibodies, peroxidase conjugated anti-mouse secondary antibodies and a bioluminescent kit. A portion of a strain-free gels is shown as loading control. Bar plots represent the mean and standard deviation of GFP levels from three independent biological replicates, which were determined by densitometry of Western blot bands using ImageJ (https://imagej.nih.gov/ij/).

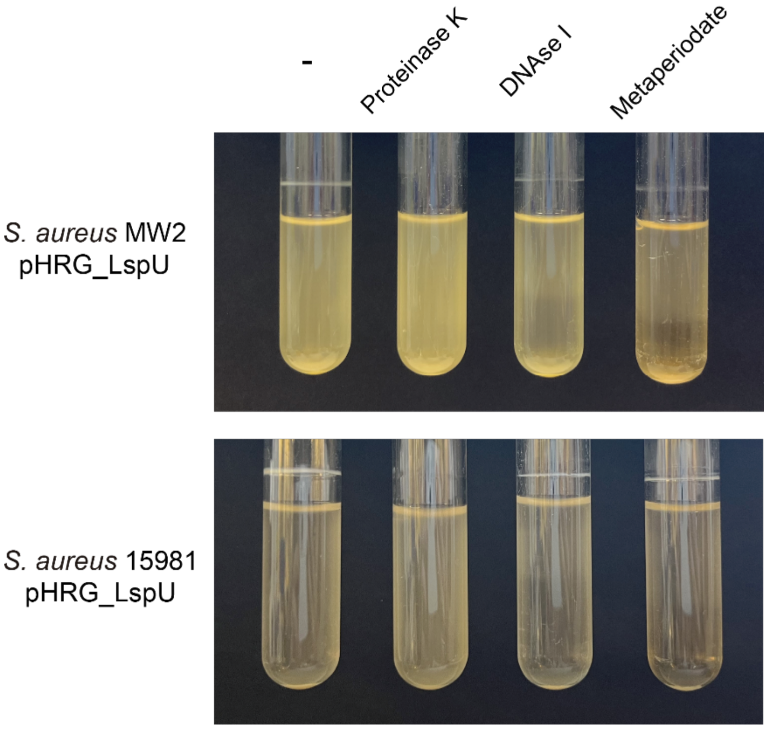

Figure S4. Biofilm characterization of LspU expressing *S. aureus* 15981 and MW2 strains. Overnight cultures grown in MH supplemented with erythromycin were treated with proteinase K (100 µg ml^{-1}), DNAse I (100 U ml^{-1}) and sodium metaperiodate (10 mM) for 2h at 37ºC with agitation and observed for any change in ring formation and/or cell aggregation in both 15981 and MW2 LspU producing strains.

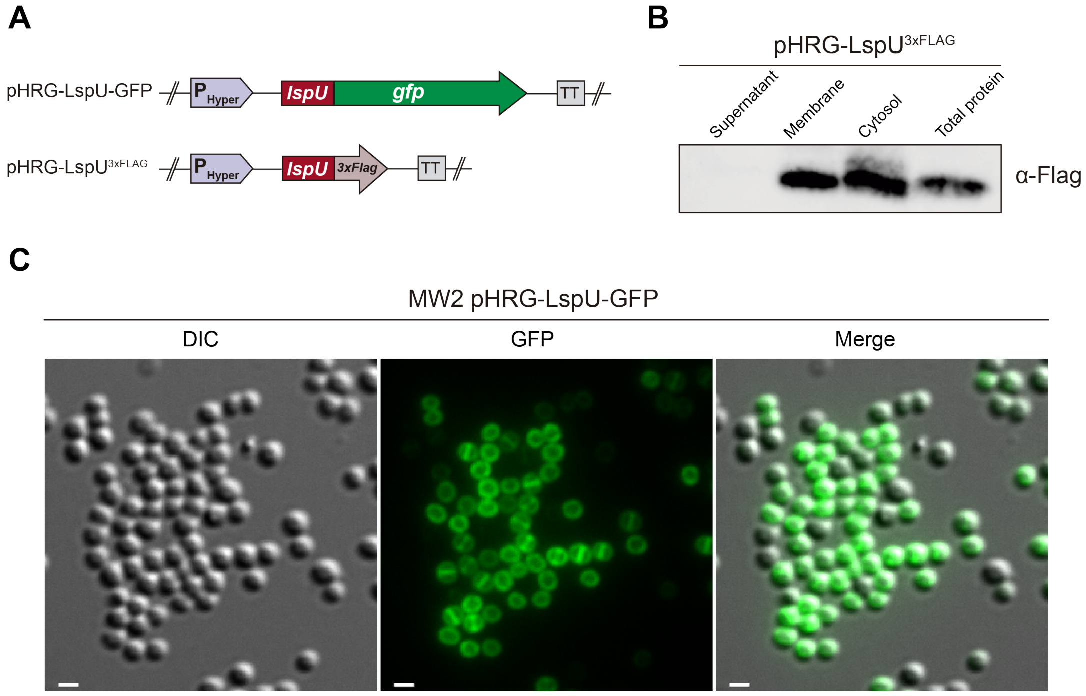

Figure S5. GFP- and 3xFLAG-tagged LspU are retained at cell membrane fractions. (**A**) Schematic representation of the constructs made to label the LspU protein with 3xFLAG and GFP. The expression of the chimeric genes is controlled by the P_{Hyper} promoter. (**B**) Western blots showing the $LspU^{3xFLAG}$ protein levels in MW2 strains carrying the pHRG-LspU-GFP and pHRG-$LspU^{3xFLAG}$ constructs in protein extract fractions from the supernatant, membrane, cytosol and total protein fractions. Membranes were developed using peroxidase conjugated anti-FLAG antibodies and a bioluminescent kit. (**C**) Representative images of microscopic acquisitions in the differential interference contrast (DIC) and green (GFP) channels of the MW2 pHRG-LspU-GFP strain. Merged DIC and GFP images (Merge) are shown. Scale bars, 1 µm.

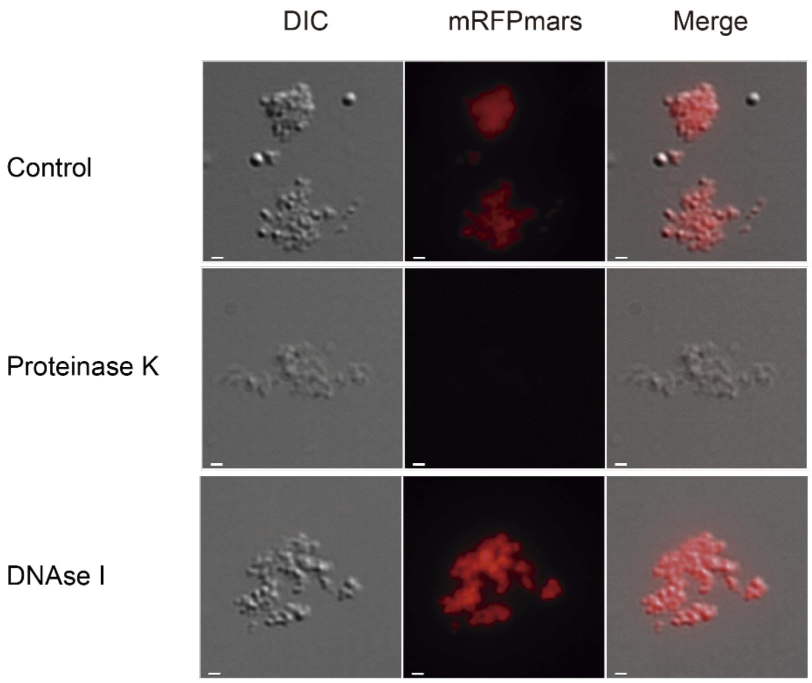

Fig S6. Proteinase K and not DNAse I degrades LspU induced cellular aggregates. Purified protein aggregates from *S. aureus* MW2 SAL2-MARS strain were treated with proteinase K (100 μg ml^{-1}), DNAse I (100 U ml^{-1}) and sodium metaperiodate (10 mM) for 2h at 37ºC. Representative images of microscopic acquisitions in the differential interference contrast (DIC) and red (mRFPmars) channels, as well as merged images (Merge) are shown. Scale bars, 1 μm.

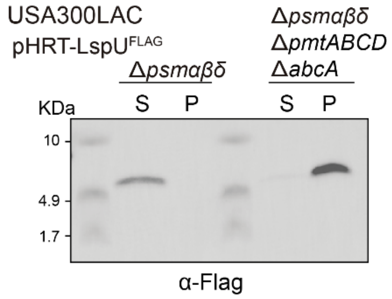

Figure S7. LspU utilizes the PSMs transporter systems. Western blots showing the LspU^{FLAG} protein levels in protein extracts from the SDS soluble (S) and cell pellet (P) fractions after treatment with 1% SDS of bacterial cells harvested from overnight cultures of *S. aureus* LAC strains, which lack PSMs- and/or PSM transporter systems-encoding genes and carry the pHRT-LspU^{FLAG} plasmid. Membranes were developed using peroxidase conjugated anti-FLAG antibodies and a bioluminescent kit.

SUPPLEMENTARY REFERENCES

1. Bastet L, Bustos-Sanmamed P, Catalan-Moreno A, Caballero CJ, Cuesta S, Matilla-Cuenca L, Villanueva M, Valle J, Lasa I, Toledo-Arana A. 2022. Regulation of Heterogenous LexA Expression in *Staphylococcus aureus* by an Antisense RNA Originating from Transcriptional Read-Through upon Natural Mispairings in the *sbrB* Intrinsic Terminator. IJMS 23:576.
2. Charpentier E, Anton AI, Barry P, Alfonso B, Fang Y, Novick RP. 2004. Novel Cassette-Based Shuttle Vector System for Gram-Positive Bacteria. Appl Environ Microbiol 70:6076–6085.
3. Mielich-Süss B, Wagner RM, Mietrach N, Hertlein T, Marincola G, Ohlsen K, Geibel S, Lopez D. 2017. Flotillin scaffold activity contributes to type VII secretion system assembly in *Staphylococcus aureus*. PLoS Pathog 13:e1006728.
4. Monk IR, Shah IM, Xu M, Tan M-W, Foster TJ. 2012. Transforming the Untransformable: Application of Direct Transformation To Manipulate Genetically *Staphylococcus aureus* and *Staphylococcus epidermidis*. mBio 3:e00277-11.
5. Valle J, Toledo-Arana A, Ghigo J-M, Amorena B, Penadés JR, Lasa I. 2003. SarA and not sB is essential for biofilm development by. Molecular Microbiology.
6. Baba T, Takeuchi F, Kuroda M, Yuzawa H, Aoki K, Oguchi A, Nagai Y, Iwama N, Asano K, Naimi T, Kuroda H, Cui L, Yamamoto K, Hiramatsu K. 2002. Genome and virulence determinants of high virulence community-acquired MRSA. The Lancet 359:1819–1827.
7. Dickey SW, Burgin DJ, Huang S, Maguire D, Otto M. 2023. Two transporters cooperate to secrete amphipathic peptides from the cytoplasmic and membranous milieus. Proc Natl Acad Sci USA 120:e2211689120.
8. Catalan-Moreno A, Cela M, Menendez-Gil P, Irurzun N, Caballero CJ, Caldelari I, Toledo-Arana A. 2021. RNA thermoswitches modulate *Staphylococcus aureus* adaptation to ambient temperatures. Nucleic Acids Research 49:3409–3426.
9. Bronesky D, Desgranges E, Corvaglia A, François P, Caballero CJ, Prado L, Toledo-Arana A, Lasa I, Moreau K, Vandenesch F, Marzi S, Romby P, Caldelari I. 2019. A multifaceted small RNA modulates gene expression upon glucose limitation in *Staphylococcus aureus*. The EMBO Journal 38:e99363.
10. Arnaud M, Chastanet A, Débarbouillé M. 2004. New Vector for Efficient Allelic Replacement in Naturally Nontransformable, Low-GC-Content, Gram-Positive Bacteria. Appl Environ Microbiol 70:6887–6891.
11. Wattam AR, Davis JJ, Assaf R, Boisvert S, Brettin T, Bun C, Conrad N, Dietrich EM, Disz T, Gabbard JL, Gerdes S, Henry CS, Kenyon RW, Machi D, Mao C, Nordberg EK, Olsen GJ, Murphy-Olson DE, Olson R, Overbeek R, Parrello B,

Pusch GD, Shukla M, Vonstein V, Warren A, Xia F, Yoo H, Stevens RL. 2017. Improvements to PATRIC, the all-bacterial Bioinformatics Database and Analysis Resource Center. Nucleic Acids Res 45:D535–D542.

12. Olson RD, Assaf R, Brettin T, Conrad N, Cucinell C, Davis JJ, Dempsey DM, Dickerman A, Dietrich EM, Kenyon RW, Kuscuoglu M, Lefkowitz EJ, Lu J, Machi D, Macken C, Mao C, Niewiadomska A, Nguyen M, Olsen GJ, Overbeek JC, Parrello B, Parrello V, Porter JS, Pusch GD, Shukla M, Singh I, Stewart L, Tan G, Thomas C, VanOeffelen M, Vonstein V, Wallace ZS, Warren AS, Wattam AR, Xia F, Yoo H, Zhang Y, Zmasek CM, Scheuermann RH, Stevens RL. 2023. Introducing the Bacterial and Viral Bioinformatics Resource Center (BV-BRC): a resource combining PATRIC, IRD and ViPR. Nucleic Acids Research 51:D678–D689.
13. Schwengers O, Jelonek L, Dieckmann MA, Beyvers S, Blom J, Goesmann A. 2021. Bakta: rapid and standardized annotation of bacterial genomes via alignment-free sequence identification. Microbial Genomics 7.
14. Madhaiyan M, Wirth JS, Saravanan VS. 2020. Phylogenomic analyses of the Staphylococcaceae family suggest the reclassification of five species within the genus Staphylococcus as heterotypic synonyms, the promotion of five subspecies to novel species, the taxonomic reassignment of five Staphylococcus species to Mammaliicoccus gen. nov., and the formal assignment of Nosocomiicoccus to the family Staphylococcaceae. International Journal of Systematic and Evolutionary Microbiology 70:5926–5936.
